# Gut Microbiota Composition and Predicted Microbial Metabolic Pathways of Obesity Prone and Obesity Resistant Outbred Sprague-Dawley CD Rats May Account for Differences in Their Phenotype

**DOI:** 10.3389/fnut.2021.746515

**Published:** 2021-12-07

**Authors:** Diana N. Obanda, Michael J. Keenan, Ryan Page, Anne M. Raggio, Christopher M. Taylor, Brian D. Marx, Rhett W. Stout, Justin Guice, Meng Luo, David A. Welsh, Diana Coulon, Claudia Husseneder

**Affiliations:** ^1^Department of Nutrition and Food Sciences, University of Maryland, College Park, MD, United States; ^2^LSU Department of Nutrition and Food Sciences/Animal Sciences, LSU AgCenter, Baton Rouge, LA, United States; ^3^LSU School of Medicine Department of Microbiology, Immunology & Parasitology, New Orleans, LA, United States; ^4^LSU Department of Experimental Statistics, LSU, Baton Rouge, LA, United States; ^5^LSU School of Veterinary Medicine, Baton Rouge, LA, United States; ^6^BIO-CAT, Troy, VA, United States; ^7^LSU Department of Entomology, LSU AgCenter, Baton Rouge, LA, United States

**Keywords:** obese prone, obese resistant, microbiota, resistant starch, fermentation

## Abstract

Like humans, outbred Sprague-Dawley CD rats exhibit a polygenic pattern of inheritance of the obese phenotype and not all individuals exposed to a high calorie intake develop obesity. We hypothesized that differences in gut microbiota composition account for phenotype differences between obese prone (OP) and obese resistant (OR) rats. We studied the gut microbiota composition of OPand OR rats after a high fat (HF) diet and how they respond to fermentation of resistant starch (RS). In phase 1 of the study 28 OP and 28 OR rats were fed a HF diet. In order to determine causal role of microbiota on phenotypes, In phase 2, a microbiota transplant between the two phenotypes was performed before switching all rats to a HF diet supplemented with 20% RS. We determined microbiota composition by 16S sequencing and predicted microbiota function by PICRUSt2. Despite a similar calorie intake, in phase 2 OP rats gained more weight and accumulated more abdominal fat in both phase 1 and 2 compared to OR rats (*P* < 0.001; *n* = 6). The OP rats fermented RS more robustly compared with OR rats with an increase in total bacteria, short chain fatty acids, and increased weight of the cecum, but microbiota of OP rats had much lower alpha diversity and evenness. The microbiota of OP rats, had higher amounts of bacteria from order Bacteroidales, specifically family Muribaculaceae (*S24-7*), which is known to possess several starch degrading enzymes and was reported in previous studies to increase with fermentation of RS. The OR rats fermented RS less but had higher bacterial diversity and evenness and had significantly higher bacterial counts from phylum Firmicutes particularl*y* order Clostridiales, genus *Clostridium* and an uncultured bacterium of the genus *Akkermansia*. The microbiota of OR rats had enhanced bacterial chemotaxis, phosphotransferase system (PTS), and fatty acid biosynthesis compared to OP rats whose microbiota had higher glycan degradation and LPS biosynthesis pathways. The microbiota transplant did not change obesity phenotype or microbiota composition. In conclusion, a higher alpha-diversity and evenness of the microbiota and higher proportions of Clostridiales and *Akkermansia* in OR rats were associated with a better metabolic phenotype with lower body fat. However, robust RS fermentation caused a lower diversity and evenness and did not result in a leaner phenotype.

## Introduction

Several studies have consistently shown that variations in bacterial types in the gut microbiota are associated with host phenotype: obese or lean. While causal implication of specific bacterial species to obesity is not clear, variation in the composition of the gut microbiota has been demonstrated (1, 2). The gut microbiota influences host phenotype through direct contact with intestinal cells or indirectly through bacterial metabolites that impact cellular mechanisms. We chose to study the microbiota composition of outbred Sprague-Dawley CD rats because they mimic the human obese phenotype in terms of a polygenic pattern of inheritance. Like humans, when these rats are exposed to excess energy with a high fat diet, not all of them develop obesity. The obesity-resistant rats (OR) retain a leaner frame while the obesity-prone (OP) rapidly gain weight within 4 weeks when fed the same high fat (HF) diet (3, 4). Because leptin production and receptor sensing in these rats is not impaired, they do not display extreme endocrine disturbances such as those in db/db mice or the Zucker diabetic fatty rats.

In addition to responding differently to a HF diet, we have previously shown that the OP and OR rats respond differently to dietary resistant starch (RS) with the OP fermenting RS robustly while the OR minimally ferment (5). However, fermentation by OP or OR rats did not result in greater body fat when compared to the OP or OR rats fed the same diets except without resistant starch (5). Thus, dietary fiber fermentation products such as short chain fatty acids (SFCA) may not be a contributary factor in the ability of OR rats to resist fat accumulation. The robust fermentation in OP rats is accompanied by increases in the family Muribaculaceae (previously known as S24-7), and the archaea *Methanobrevibacter smithii*, which are both markers of fermentation of RS (5).

Studies examining the role of the gut microbiota in relation to obesity have consistently shown a lower relative abundance of phylum Bacteroidetes and a higher relative abundance of phylum Firmicute*s* in obese mice and humans compared to lean subjects (1, 2). However, knowledge on the role of specific bacterial species in causation of the obese phenotype is minimal. It is possible that variations in specific bacterial species rather than phylum level changes are more important. For example, more recent studies have shown that some genera among Firmicutes impact host obesity phenotype by acting through the intestinal immune system. Specifically, abundance of class Clostridia increases IgA secretion, host lipid absorption by modulating the expression of CD36 a protein involved the transport and absorption of fat in the small intestines, and overall intestinal immune phenotype (6).

**Figure 1:**
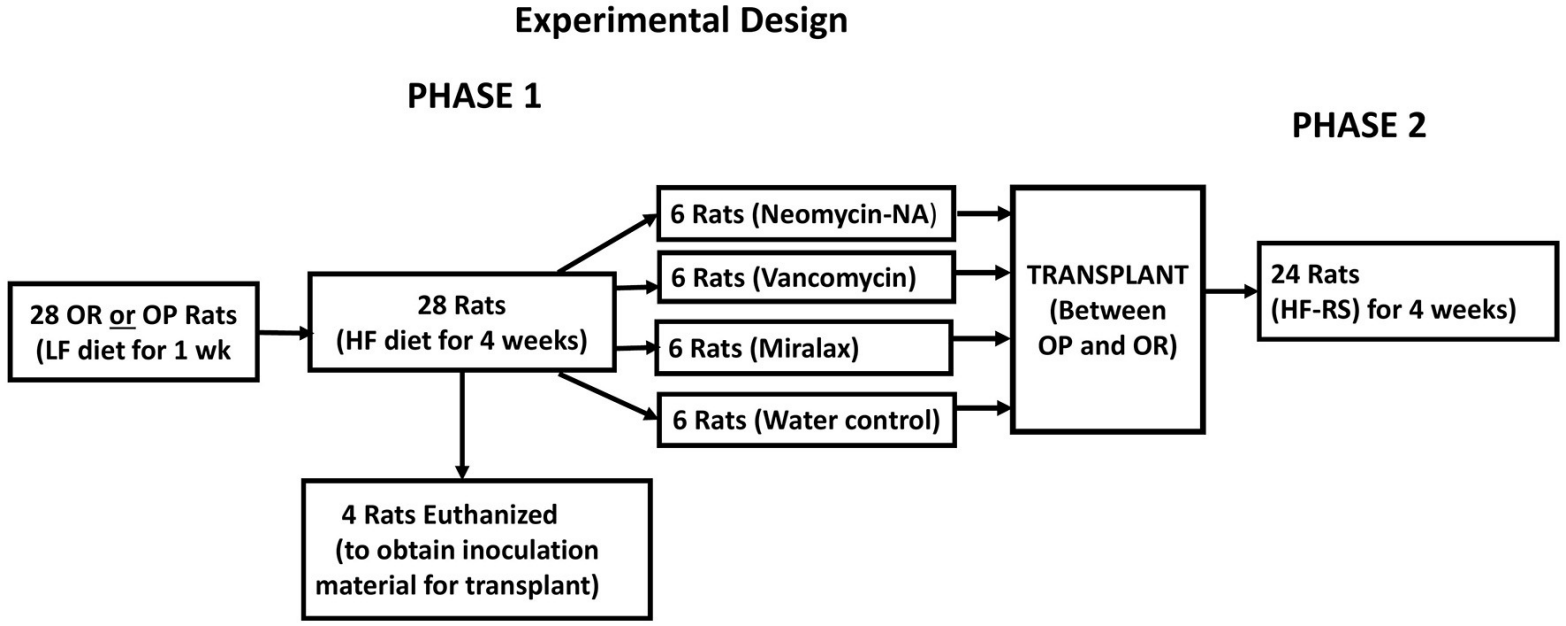
Flow diagram of the Experimental design.

The gut microbiota of obese humans and animals are characterized by reduced species richness and diversity and this may impact metabolic capacity (1, 2). Maintenance of diversity and collective functional capacity of the microbiota is important for optimal health. The richness and diversity of the gut microbiota in OP and OR rats have not been evaluated. We hypothesized that differences in the obesity phenotype may be attributed to the microbiota composition, differences in the richness, and overall diversity of bacterial species and in metabolic function of the bacteria. Herein we report findings from a feeding study that includes a HF diet and a HF diet supplemented with resistant starch and a fecal transplant between the two phenotypes (Figure 1). Prior to the transplant, we tested the efficacy of two antibiotic formulations and MiraLAX® in reducing the gut bacterial load in the recipient rats to create a niche for the probiotic bacteria to establish effectively (7). MiraLAX® is an over-the-counter colonoscopy preparation that knocks down bacterial loads in the lower gut (8). We compared its efficacy to that of the antibiotics in creating a niche for the probiotic bacteria transplant. We report on the effects of diet, antibiotics, MiraLAX® and the transplant on microbiota composition, alpha diversity, beta diversity, and microbial function in relation to the obesity phenotype.

## Materials and Methods

### Animals and Study Plan

The study was approved by the Louisiana State University Institutional Animal Care and Use Committee (IACUC) as 15–109. After 1 week of quarantine, 4-week-old male 28 OP and 28 OR rats (Charles River Laboratories) were singly housed in a temperature-controlled vivarium with a 12:12-h light/dark cycle and acclimated to a powdered low-fat diet for a week.

### Phase 1 Procedures

Rats were fasted (~6 h) before blood collection by retro-orbital bleeding to determine serum glucose and insulin levels for calculation of the homeostatic model of assessment of insulin resistance index (HOMA-IR). A flow diagram of the study design is shown in Figure 1. All OP and OR rats were then fed the HF diet for 4 weeks (phase 1). Food and water were provided *ad libitum*. Body weight and food intake were determined twice a week. The composition and energy value of the HF diets is shown in Table 1.

**Table 1:** Diets for obesity prone and obesity resistant rats.

**Diet components**		**Phase 1 diet**	**Phase 2 diet**
**Ingredients (g)**	***Energy value (Kcal/g)***	**HF (g)**	**HF-RS (g)**
^a^AMIOCA™ starch	3.5	405.7	31.8
^b^HI-MAIZE® 260 RS	2.8	0	472.4
Sucrose	4.0	100	100
Casein	3.50	140	140
Cellulose	0	106.2	7.7
Corn oil	8.84	100	100
Lard	9.00	100	100
Mineral mix (AIN-93M)	0.88	35	35
Vitamin mix (AIN 93)	3.87	10	10
Choline chloride	0	1.3	1.3
L-Cystine	4	1.8	1.8
Total weight (Kcal)		1,000 (4182.5)	1,000 (4182.7)

a *AMIOCA™ is a 100% amylopectin cornstarch product from Ingredion (Bridgewater, NJ)*.

b *HI-MAIZE® 260 is high-amylose cornstarch from Ingredion Incorporated (Bridgewater, NJ). The batch used was 42.3% resistant starch based on wet weight for use in diet*.

After feeding all rats a HF diet in phase 1, four OP, and four OR rats were euthanized for determination of abdominal body fat accumulation and collection of cecal contents as inoculum for the transplant. Procedures for euthanasia, collection and processing of tissues are similar to those shown in Obanda et al. (9). Cecal contents of the 8 rats euthanized were collected by tying off the cecum before removal from the GI tract aseptically and aerobically in a hood blowing filtered air, diluted 1:10 with sterile 15% glycerol/PBS solution. The diluted cecal contents from the four OP rats were pooled into one sample and those from the four OR rats were also pooled into one sample and stored at −80°C.

### Phase 2 Procedures

The remaining 24 OP and 24 OR rats were separately divided into 4 sub-groups of 6 each stratified using body weight and HOMA-IR. A sample size of *n* = 6 for each group was used based on a power analysis in a previous resistant starch fermentation study that showed 6 to be sufficient to attain a statistical power of 0.90 for a one tailed test and 8 samples for a two tailed test (10). Fresh fecal samples were collected from under the feeding cages for bacterial load determination prior to a knock down treatment.

The knockdown and transplant treatments performed separately for OP and OR rats were as shown below:

1. Sub-group 1-broad-spectrum antibiotics neomycin + ampicillin (NA) at a dose of 55 mg/kg/day for neomycin and 110 mg/kg/day for ampicillin by gavage once daily for 3 days.
2. Sub-group 2-one narrow-spectrum and one broad-spectrum antibiotic: vancomycin and meropenem (VM) both at a dose of 50 mg/kg/day by gavage once daily for 3 days.
3. Sub-group 3: MiraLAX® (LX) a colonoscopy preparation tested as an alternative to antibiotics. After a water gavage for 2 days, MiraLAX® was given on the 3rd day in two gavages 3 h apart at a dose of 63 mg in 437 ul for a 250 g rat calculated based on a human dose of 17 g dissolved in 4–8 ounces for a 150-lb. person. All other groups were given an additional gavage of water to match the stress of an additional gavage in the MiraLAX group.
4. Sub-group 4: gavaged with water (WA) as control treatment once daily for 2 days and twice on the third day.

After knock-down treatments, rats had free access to HF diet and water for 2 days. Fresh fecal samples were collected from under the feeding cages within 24 h after knockdown treatments.

Three days after knockdown, each of the OP rats was gavaged with one ml aliquot of cecal contents pooled from 4 donor OR rats. Similarly, each of the OR rats was gavaged with one ml of contents pooled from 4 donor OP rats. Rats given water as control for knock-down again received water as transplant control. After transplants, the rats were switched to the high fat diet containing resistant starch (HF-RS) and fed *ad libitum*. The composition and energy value of the HF-RS diet is shown in Table 1. It was formulated to contain 20% high-amylose cornstarch (HI-MAIZE® 260 RS) by weight and was isocaloric to the HF diet. AMIOCA™ powder corn starch has 100% digestible amylopectin. Food uptake and body weight were monitored for another 4 weeks.

### Euthanasia and Collection of Tissues

After 4 weeks of phase 2, rats were euthanized by isoflurane inhalation. Excised retroperitoneal, perirenal, and epidydimal fat pads were used to determine total abdominal fat weight. The weight of the gastrointestinal tract without contents was added to the disemboweled body weight to obtain the emboweled body weight (EBW). Cecal contents were snap frozen in liquid nitrogen and stored at −80°C.

### Bacterial DNA Extraction and Quantification of 16S rRNA Gene Copies by QPCR

Genomic DNA from fecal samples and cecal contents was extracted using the QIAamp DNA stool MiniKit (Qiagen, Valencia, CA) with a modified method including a bead-beating process. About 180–220 mg of each sample was mixed with 0.2 g zirconia/silica beads (diameter: 0.1 mm, Biospec) and lysis buffer. RNase A was added to remove RNA before passing the eluate through a concentration column. Quality and concentration of the purified DNA were determined using a NanoDrop 1000. Isolated DNA was stored at −20°C and used for 16S amplicon library preparation or qPCR.

To quantify the 16S gene in fecal contents, the universal primers F:5′ACGTCRTCCMCNCCTTCCTC3′ and R:5′GTGSTGCAYGGYYGTCGTCA3′targeting the V2-V3 regions of the 16S gene as shown before in Belenguer et al. (11), were prepared by Integrated DNA Technologies (Coralville, Iowa). The Basic Local Alignment Search Tool (https://blast.ncbi.nlm.nih.gov/Blast.cgi, accessed June 26th, 2017, was used to verify *in silico* that these primers for the universal 16S rRNA gene matched a broad range of bacteria. The SYBR Green qPCR assay was used to quantify the 16S gene on the ABI Prism 7900HT Sequence Detection System (Life Technologies, Foster City, California). Cycling conditions were 50°C for 2 min, 95°C for 10 min, and 40 cycles of 95°C for 15 s, followed by primer annealing at 60°C and a final 78°C for 30 s. Amplicon quantities (gene copies per microliter) vs. cycles-to-quantification standard curves were used to determine total bacteria (16S rRNA gene) quantities. Molar concentrations of amplicon standard DNA were converted into gene copies per gram cecal contents as shown in Obanda et al. (9).

### 16S rRNA Gene Sequencing

Amplification, sequencing, and bioinformatics of bacterial DNA from phase 2 were performed at the LSU Microbial Genomics Resource Group. Briefly, the 16S rRNA gene hypervariable region V4 in 20 ng of DNA was amplified using gene-specific primers were F:5′TCGTCGGCAGCGTCAGATGTGTATAAGAGACAGGTGCCAGCMGCCGCGGTAA3′ R:5′GTCTCGTGGGCTCGGAGATGTGTATAAGAGACAGGGACTACHVGGGTWTCTAAT 3′ as shown in Walters et al. (12). PCR conditions were 95°C for 3 min, 25 cycles of 95°C for 30 s, 55°C for 30 s and 72°C for 30 s, 72°C for 5 min and holding at 4°C. Two steps of amplification were performed to prepare the sequencing library using AccuPrime Taq high fidelity DNA polymerase system (Invitrogen). A negative control from DNA extraction and a positive control from Microbial Mock Community HM-276D (BEI Resources) were included during library preparation. PCR products were purified using AMPure XP beads. Two μl of purified amplicon DNA was amplified for 8 cycles with the same PCR conditions using primers with different molecular barcodes: forward 5′ AATGATACGGCGACCACCGAGATCTACAC (i5) TCGTCGGCAGCGTC 3′; reverse 5′ CAAGCAGAAGACGGCATACGAGAT (i7) GTCTCGTGGGCTCGG 3′. Expected amplicon length is ~290–294 bp including the specific primer. The indexed amplicon libraries were purified using AMPure XP beads, quantified by Quant-iT PicoGreen (Invitrogen) and pooled together. The pooled library was quantified using a KAPA Library Quantification Kit (Kapa Biosystems), diluted, and denatured as per the guideline of the Illumina MiSeq sequencing library preparation. Paired-end sequencing (2 × 250 bp) was performed on a MiSeq (Illumina) using MiSeq Reagent Kit v2 (500-cycles).

### 16S rRNA Gene Amplicon Sequencing Analysis

Fastq files from the Illumina MiSeq run were processed through the QIIME2 pipeline as shown by Bolyen et al. (13) using the DADA2 plugin (14) to determine amplicon sequence variants (ASVs). ASVs are a collection of exact sequences having a defined statistical confidence and are generated without clustering or reference databases. Quality control and trimming of raw reads were performed with parameters set based on quality profiles of the sequencing run to retain high quality data and trim off amplicon primers. The DADA2 algorithm requires a uniform truncation length across all reads and was used to build an error profile for the samples. Quality scores were above 25 until the last 10 bp so forward and reverse reads were truncated at length 240 bp and primer sites were removed. Forward and reverse reads were merged to produce full amplicons and resulting amplicons outside of the expected 250–254 bp range were filtered out followed by chimera detection and removal as detailed in Callahan et al. (14). Data was rarefied to a sampling depth of 10,000 reads per sample. Any ASVs that appeared in only one sample were removed and the remaining ones were aligned using mafft and a phylogenetic tree was built with FastTree. Taxonomic classification was performed using the SILVA v138 database (15). We performed multivariate testing between treatment groups and further performed *post-hoc* comparisons when significant differences were observed. We determined alpha diversity indices: ASV's (a count of unique ASV's in each sample), Pielou's evenness (a measure of how close in numbers the species in a community are), Shannon diversity (to account for both abundance and evenness of the sequence variants present), and Faith pd which uses phylogenetic distance (branch length of tree) to calculate the diversity in an individual sample. Beta diversity was calculated based on the weighted unifrac distance metric and visualized using principal component analysis (PCA) as implemented in QIIME2.

### Linear Discriminant Analysis

Using the Galaxy online workflow application (https://huttenhower.sph.harvard.edu/galaxy), we determined the taxa most likely to explain differences between the OP and OR rats using linear discriminant analysis (LDA) effect size (LEfSe). LEfSe utilizes a non-parametric Kruskal-Wallis rank sum test to assess differential features with significantly different abundances between assigned taxa and performs LDA to estimate the effect size of each sequence variant as reported by Segata et al. (16). LDA scores ranking differential taxa are displayed on a LEfSe bar chart according to their effect size. For LEfSe analysis data are first converted to log_10_ before the non-parametric Kruskal-Wallis rank sum test. A significant alpha level of 0.05 and an effect size threshold of a three times greater difference was used for displaying results in this study.

### PICRUSt2

To predict the metabolic pathways, phylogenetic investigation of communities by reconstruction of unobserved states (PICRUSt2) was performed from sequencing data, as shown before (17). Fastq sequence files for each sample were processed using QIIME2. Representative sequences were used as input files for the PICRUSt2 analysis pipeline. Metabolic pathways were assigned based on the Kyoto Encyclopedia of Genes and Genomes (KEGG) Ortholog (KO) database. Read abundance data for all predicted pathways were converted to relative abundance (%) and by the Galaxy server (https://huttenhower.sph.harvard.edu/galaxy/) for LEfSe analysis using LDA score 3.0 as a threshold level to determine pathways taxa most likely to explain differences between the OP and OR rats.

### Statistical Analyses

Data for phase 1 were analyzed by *t*-tests in SAS 9.4 by the PROC TTEST. At the end of the study, phase 2 data were analyzed in SAS 9.4 by the PROC TTEST to compare effects of phenotype (OP vs. OR while the effects of the treatments (VM, NA, LX, WA) were compared by PROC GLM test (SAS Institute, Cary, North Carolina). Effects of the knockdown treatments and of the transplant were compared to those of the water control. Main and interactive effects were considered significant at *P* < 0.05 and expressed as means ± SE. Further tests were performed using the Tukey's test of multiple comparisons. Data were analyzed for outliers using studentized residuals. Statistical significance for analysis of alpha and beta-diversity was determined with a variety of tests in QIIME2: the multiway Adonis test of differentiation among microbiota based on location and spread of samples followed by Permdisp to determine pairwise dispersion (spread of samples) and pairwise tests for differentiation in centroid location (Permanova) after 999 permutations. The Spearman and Pearson correlations were performed between ECW and diversity or relative abundance of particular taxa using the QIIME2 output. The relative abundance values of different taxa were converted to percentages.

## Results

### Energy Intake, Body Weight, Body Fat, and Fermentation Variables

Although all animals were fed the same diets with no differences in energy intake as shown by end of phase 2, OP rats gained more body weight, had higher fat accretion (*P* < 0.0001) in both phases 1 and 2 compared to the OR rats. During phase 1 (1st 4 weeks), all animals were fed a HF diet. Phase 2 (2nd 4 weeks) began after the microbiota transplant between the OP or OR rats and then the rats were switched to a HF diet containing RS. The OP rats had higher body weight, abdominal fat weight and EBW compared to OR rats. The knockdown type and transplant had no effect on body weight, abdominal fat weight and EBW (Tables 2, 3). The OR rats given the NA treatment had lower abdominal fat than other groups among OR. The pH and quantity of SCFAs in cecal contents were not determined in phase 1 because all the cecal contents from the rats euthanized were used for the transplant. In phase 2 after the knockdown and transplant and including resistant starch in the diet, the empty cecum weight, and total amount of SCFAs (acetate, butyrate, and propionate) were higher in OP rats while pH of the cecal contents was lower in OP rats (Tables 2, 3). Only phenotype impacted the amounts of SCFAs, pH of cecal contents and weight of the cecum reflecting higher fermentation in OP rats (Table 3). The transplant after treatment with antibiotics or MiraLAX had no effects on fermentation parameters compared to the water controls in both OP and OR rats (Table 3). After the robust fermentation of RS in OP rats during Phase 2, they had relatively higher amounts of total bacteria compared to OR rats which had modest fermentation as determined by quantifying the 16S rRNA gene (Tables 2, 3; *P* < 0.05; *n* = 6). The rats from either phenotype had greater amounts of bacteria with the MiraLAX® knockdown treatment.

**Table 2:** Body weight, Body fat, and Fermentation variables at the end of Phase 1.

	**OP means (SE)**	**OR means (SE)**	***P*-value after *T*-Test**
Energy intake (Kcal/d)	81.95 (0.76)	78.17 (1.07)	ns
Body weight (g)	378.1 (13.2)	303.1 (8.47)	<0.01
EBW (g)	369.4 (10.3)	292.01 (8.2)	<0.01
Abdominal fat (g)	18.9 (1.29)	10.8 (0.70)	<0.001
Full cecum weight (g)	2.74 (0.51)	2.56 (0.73)	ns
Empty cecum weight (g)	0.70 (0.13)	0.73 (0.04)	ns
Total SCFAs (g)	–	–	–
pH of cecal contents	–	–	–
Total Bacteria per g fecal sample (X 10^9^ gene copies of 16S gene)	1.04 (0.04)	1.64 (0.24)	<0.01

**Table 3:** Body weight, Body fat, and Fermentation variables at the end of Phase 2.

	**OP means (SE)**				**OR means (SE)**				***P*-value** **After ANOVA**
	**VA**	**NA**	**LX**	**WA**	**VA**	**NA**	**LX**	**WA**	
Energy intake Kcal/d)	83.4 (4.01)	76.2 (3.08)	74.6 (1.62)	78.1 (3.62)	77.6 (1.89)	74.1 (2.87)	78.0 (1.92)	77.0 (1.18)	ns
Body weight (g)	477.3 (11.3)^a^	470.4 (13.9)^a^	454.5 (16.3)^a^	460.8 (19.5)^a^	371.7 (13.4)^b^	360.1 (11.0)^b^	359.8 (6.01)^b^	361.9 (5.72)^b^	<0.001
EBW (g)	464.8 (27.6)^a^	456.9 (33.6)^a^	445.8 (50.0)^a^	448.8 (47.1)^a^	370.1 (31.7)^b^	351.2 (25.6)^b^	359.8 (15.5)^b^	359.9 (16.6)^b^	<0.001
Abdominal fat (g)	26.4 (1.77)^a^	26.2 (2.13)^a^	25.1 (3.54)^a^	26.3 (3.94)^a^	11.2 (1.90)^b^	8.35 (1.17)^c^	10.6 (1.61)^b^	9.84 (3.94)^b^	<0.001
Full Cecum weight (g)	12.3 (0.67)^a^	12.2 (1.48)^a^	11.76 (1.35)^a^	12.64 (1.51)^a^	3.36 (0.19)^b^	3.62 (0.28)^b^	3.19 (0.25)^b^	3.80 (0.64)^b^	<0.001
Empty Cecum weight (g)	1.36 (0.07)^a^	1.28 (0.1)^a^	1.43 (0.12)^a^	1.51 (0.20)^a^	0.75 (0.03)^b^	0.87 (0.07)^b^	0.83 (0.06)^b^	0.85 (0.07)^b^	<0.001
Total SCFAs (nmole/cecum)	0.59 (0.06)^a^	0.87 (0.04)^a^	0.65 (0.04)^a^	0.61 (0.03)^a^	0.10 (0.01)^b^	0.15 (0.01)^b^	0.12 (0.01)^b^	0.14 (0.02)^b^	<0.001
pH of cecal contents	5.64 (0.8)^a^	5.78 (0.1)^a^	5.65 (0.2)^a^	5.89 (0.23)^a^	6.9 (0.12)^b^	7.2 (0.24)^b^	6.8 (0.56)^b^	6.9 (0.21)^b^	0.005
Total Bacteria per g cecal samples (X 10^8^ gene copies of 16S gene)	1.4 (0.03)^a^	1.1 (0.02)^a^	1.6 (0.04)^b^	1.0 (0.03)^a^	0.73 (0.03)^c^	0.72 (0.006)^c^	1.09 (0.03)^a^	0.66 (0.01)^c^	<0.01

*VA, Vancomycin; NA, Neomycin and Ampicillin; LX, MiraLAX® WA, Water; EBW, Embowelled body weight. P-value is for overall test (ANOVA), Different letters indicate significant differences after Tukey's test of multiple comparisons*.

### Bacterial Diversity Metrics

#### Alpha-Rarefaction

**Figure 2:**
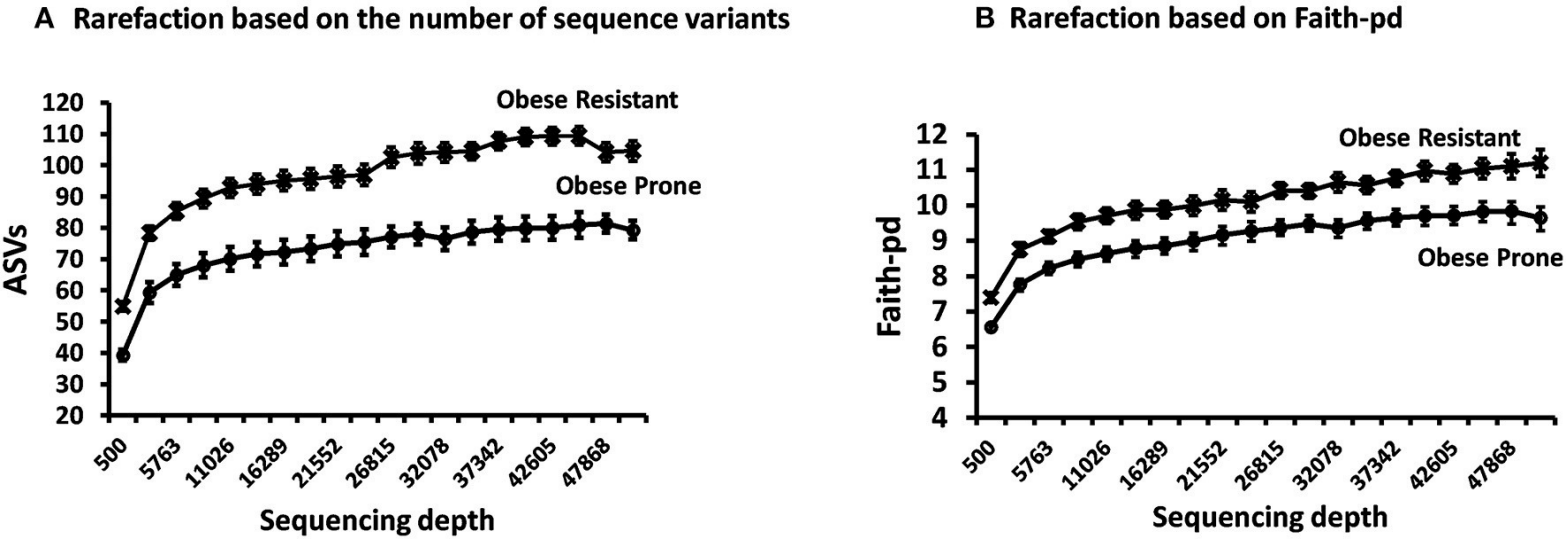
Rarefaction curves on samples obtained after phase 2. **(A)** Rarefaction based on the number of sequence variants showed richness was higher in OR rats. **(B)** Rarefaction based on Faith-Pd showed that phylogenetic distance was higher in OR rats. In both cases *n* = 24.

The rarefaction curves for all rats reached a plateau indicating that the sequencing depth was sufficient to detect majority of ASVs in each sample and capture the microbial diversity (Supplementary Figure 1). Rarefaction curves comparing phenotype indicate that the microbiota of OR rats had higher richness (more ASVs) compared to OP rats (Figure 2A) regardless of sequencing depth. Faith-pd which takes phylogenetic distance into account and is confounded by richness was also higher in OR rats (Figure 2B). The asymptotic shape of the curves in both indices indicate that a moderate sequencing depth (ca. 10,000) was sufficient to capture the majority of the diversity in each group.

#### Alpha Diversity and Evenness Measures

**Figure 3:**
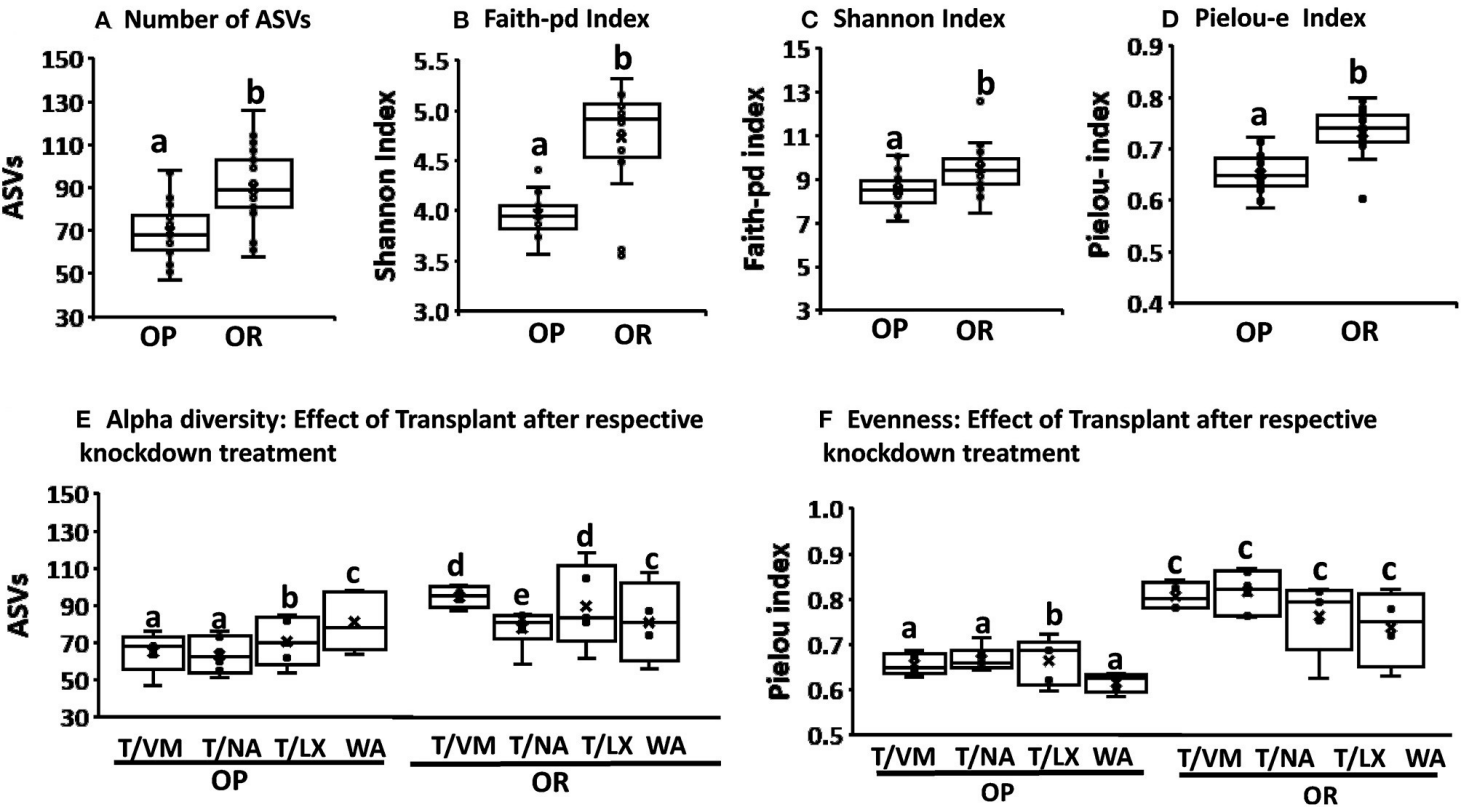
OR rats had a higher alpha diversity and eveness irrespective of the transplant. **(A–D)** Number of ASVs, Faith-pd, Shannon, Pielou-e indices were all higher in OR rats (*P* < 0.001;*n* = 6). **(E)** In rats treated with VM, NA and LX before the transplant, the number of ASVs were significantly higher in OR rats compared to OP rats (*P* < 0.02) but in rats treated with water there was no difference between OP and OR. T/LX in OP vs. T/LX in OR: *P* < 0.05; T/NA I OP vs. T/NA in OR: *p* = 0.02; T/VM I OPvs. T/VM in OR: *p* = 0.014. T/WA in OP vs. T/WA in OR: *p* = 0.8. Among OR rats, those treated with NA had lower diversity (*P* < 0.05). In all cases *n* = 6. **(F)** Pielou-e eveness index was lower in OP rats treated with WA control compared to those treated with antibiotics and LX. It was not different among all treatments in OR rats. Overall OR rats had significantly higher eveness compared to OP rats irrespective of the knockdown and transplant treatment (T/NA in OP vs. T/NA in OR: *P* < 0.004, T/VM in OP vs. T/VM in OR: *p* = 0.014; T/WA in OP vs. T/Wain OR: *p* = 0.015; T/LX in OP vs. T/LX in OR: *P* < 0.03). In all cases *n* = 6. Different letters indicate significant differences after a T-test or Tukey's test of multiple comparisons.

The composition of the microbiota and alpha and beta diversity was compared based on phenotype (OP vs. OR rats), based on knockdown method (VM, NA, LX, or WA control) and also based on the microbiota transplant outcome between the two phenotypes compared to the WA control transplant. The microbiota of OR rats had significantly higher alpha diversity with number of ASVs, Faith pd, Pielou-e measure of evenness, and Shannon indices showing significantly higher values than those of OP rats (*P* < 0.001) (Figures 3A–D). Further analysis showed that the diversity in OP and OR rats were not different in rats treated with water as control knockdown treatment (*P* = ns) but in antibiotic and MiraLAX® groups, OR rats also had higher diversity (*P* < 0.05) (Figure 3E). The OR rats also had higher diversity irrespective of the transplant (Figure 3F) indicating that the knockdown treatment and transplant did not change the microbiota. The relatively higher overall diversity in OR compared to OP rats was mainly observed in the NA and VM antibiotic treated rats. Analysis based on transplant method did not show differences between the control water transplant and the microbiota transplant in both OP and OR rats. The comparison between knockdown treatments and the transplant was made on the recovered microbiota (4 weeks after antibiotics, MiraLAX® or water treatment). Evenness as measured by the Pielou's index was higher in all OR rats compared to OP rats irrespective of knockdown and transplant. While evenness was lower in OP rats treated with water compared to those treated by NA and VM and LX, evenness was not different among all OR rats irrespective of knockdown or transplant (Figure 3F).

#### Beta Diversity Measures

**Figure 4:**
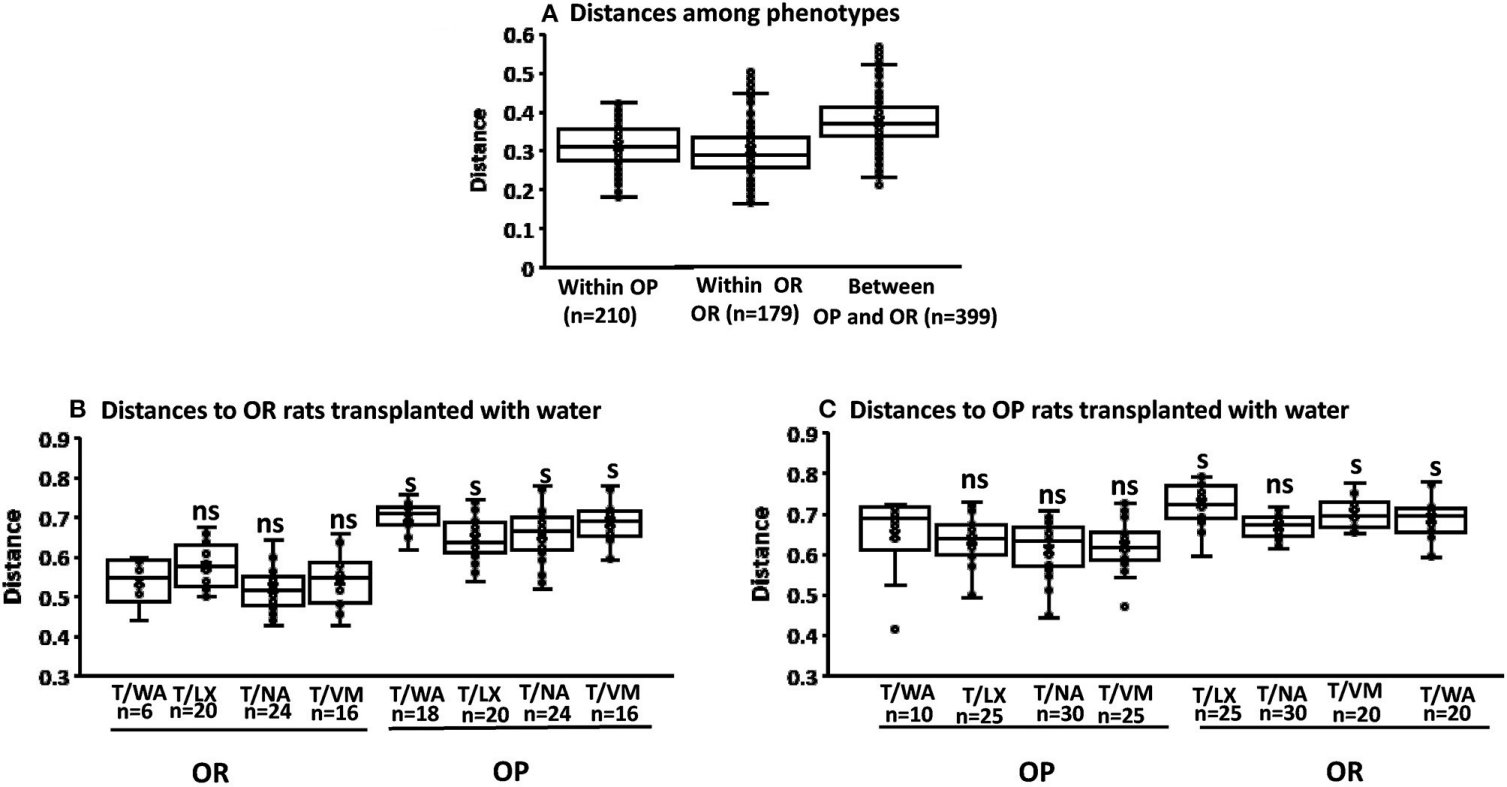
Permdisp to assess beta diversity. The first boxplot in each figure is the intra group variability, all others are inter-group variability. **(A)** No significant differences between OP and OR phenotypes (*pseudo-F* = 1.15; *P* = 0.25 *n* = the number of pairwise comparisons as shown on x-axis. **(B)** Significance of the distance to OR rats treated with water as knockdown and transplant from all other knockdown-transplant treatments (ns, not significant) (s, significant) (*P* < 0.05 after the number of pairwise comparisons shown on x-axis (*n*). Significant differences were based only on phenotypes (pseudo-*F* = 1.74 *P* = 0.02). **(C)** Significance of the distance to OP rats treated with water as knockdown and transplant from all other knockdown-transplant treatments (ns, not significant) (s, significant) (*P* < 0.05 after the number of pairwise comparisons shown on x-axis (*n*). Significant differences were based only on phenotypes (pseudo-*F* = 1.66 *P* = 0.22).

The multiway Adonis test for effects of phenotype gave an *R*^2^-value of 0.155 and *P* < 0.001 indicating that 15.5% of the variation in distances is explained by phenotype. Adonis test for effects of knockdown gave an *R*^2^-value of 0.088 indicating a smaller but significant effect size of 8% (*P* = 0.032). The interaction of phenotype and knockdown had an *R*^2^ of 0.088 and *P* = 0.036. The Adonis test measures differentiation among microbiota based on location and spread of samples defined by their community similarity in the multivariate space. No significant differences in within group variation (dispersion) of bacteria community similarities were detected for phenotype or knockdown transplant (*pseudo-F* = 1.15; *P* = 0.25 and *F* = 1.66; *P* = 0.22, respectively; weighted unifrac Permdisp (Figure 4).

**Figure 5:**
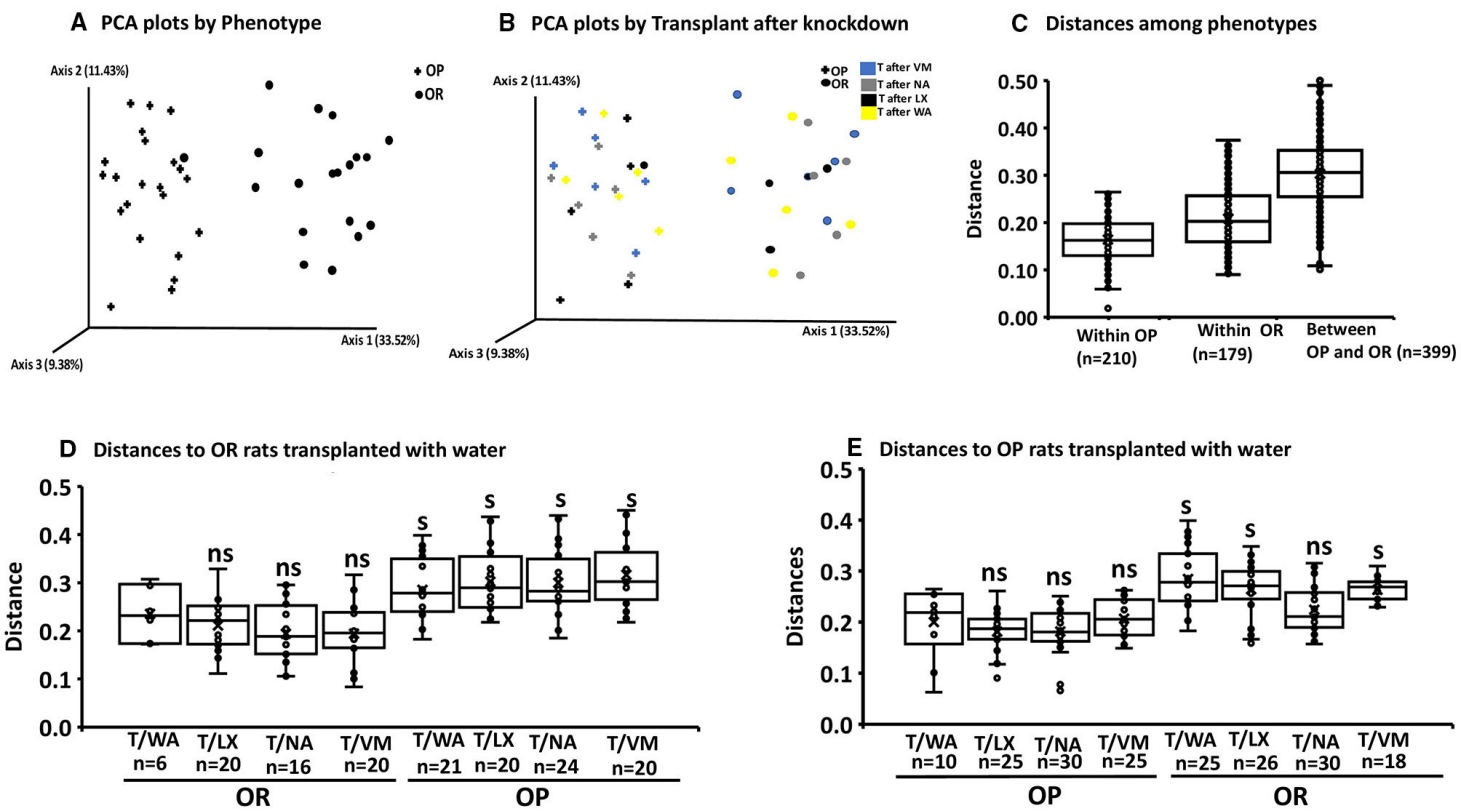
Permanova and PCA plots to assess beta diversity. The first boxplot in each figure is the intra group variability, all others are inter-group variability. Distances represent similarity of the microbial composition between the samples: **(A)** Distances based on phenotype OP vs. OR (*n* = 24). **(B)** Knock down and transplant treatment (T) did not result in group separation other than by phenotype (*n* = 6). (Example: T after VM: means Transplant with microbiota of opposite phenotype after knockdown with VM). Data points represents the distribution of principal components in 3 different directions and the closer the data points, the closer the relationship between the samples. **(C)** Significant differences between OP and OR (*pseudo-F* = 27.6; *P* = 0.001 after 399 pairwise comparisons. **(D)** Significance of the distance to OR control rats treated with water from all other knockdown-transplant treatments (*pseudo-F* = 5.34; *P* = 0.001 (ns, not significant) (s, significant) (*P* < 0.05 after the number of pairwise comparisons shown on x-axis (*n*). **(E)** Significance of the distance to OP control rats treated with water from all other knockdown treatments (*pseudo-F* = 5.34; *P* = 0.001 (ns, not significant) (s, significant) (*P* < 0.05 after the permutations shown on x-axis. (ns, not significant) (s,significant) (*P* < 0.05) after the number of pairwise comparisons shown on x-axis (*n*).

We performed additional pairwise tests for differentiation in centroid location, i.e., the mean position of all the samples within each group (Permanova). The one-way Permanova non-parametric test of significant difference between groups, based on weighted unifrac distance showed significant differences between OP and OR bacteria composition (pseudo-*F* = 27.6; *P* < 0.001). Calculated distances among the OP group, within the OR group and between OP and OR are shown in Figure 5. Figures 5A,B, visualizes them in the PCA plot. Calculated distances for knockdown treatments based on distance from the control water treated rats (WA) (Figures 5C–E). showed significant differences (pseudo-*F* = 5.34; *P* < 0.001) between microbiota composition based on phenotypes but not knockdown treatment. Tests based on transplant also showed significant differences (pseudo-*F* = 10.54; *P* < 0.001) between microbiota composition by phenotype but not transplant. The PCA plots shown in Figures 5A,B show that group separation was all based on only phenotype while knock down and transplant did not result in group separation except for one OR rat treated with LX which grouped with OP rats.

### Comparative Analysis of the Gut Microbiota Taxa Composition

**Figure 6:**
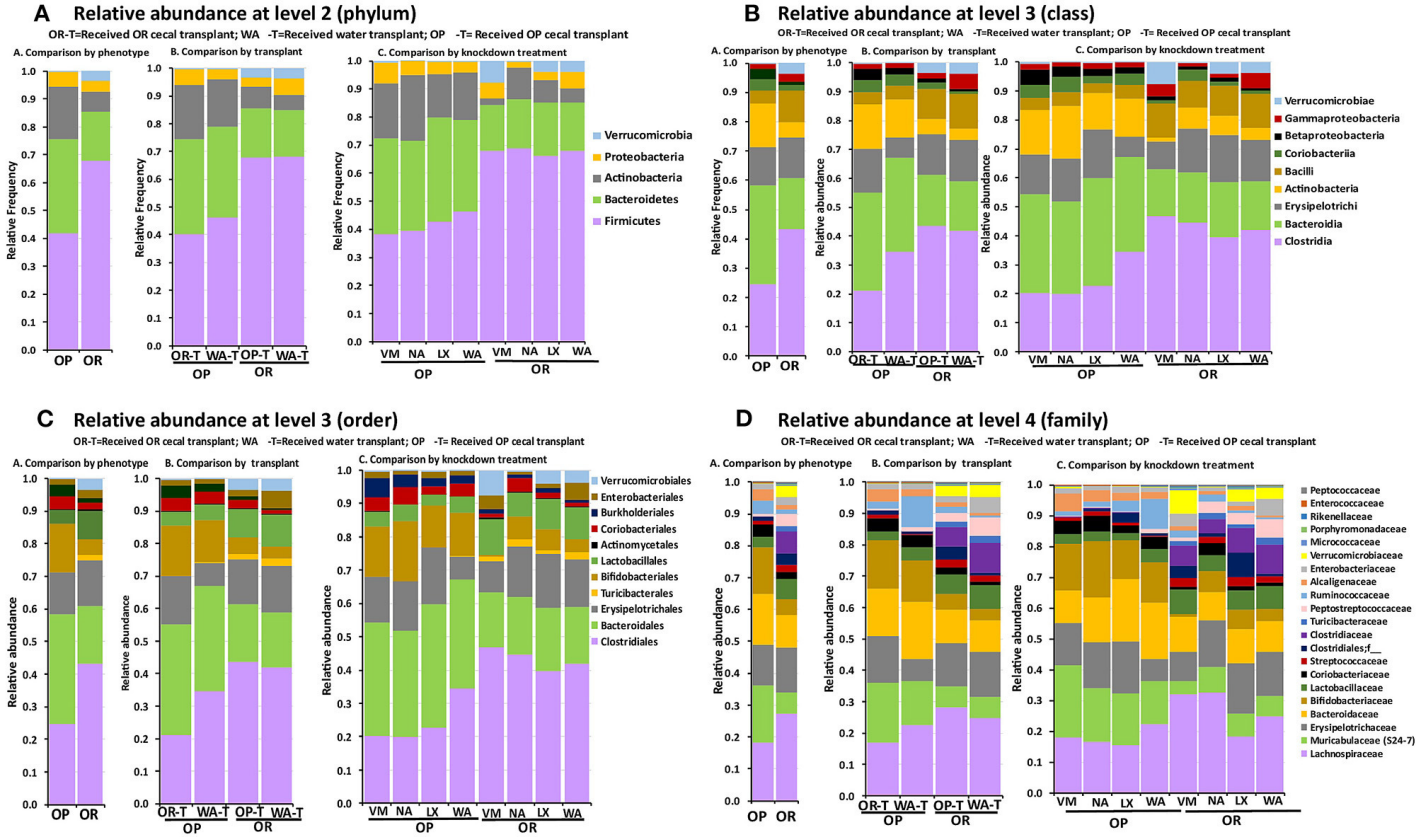
The relative abundance of bacterial groups depended only on rat phenotype but not knockdown method or the transplant. 16S sequencing data showed that the knockdown and transplant did not change the proportions of taxa between the OP and OR phenotypes. In all cases *n* = 6. **(A)** OR rats had higher amounts of phylum Firmicutes and Verrucomicrobia while OP rats had higher Bacteroidetes, Actinobacteria, and Proteobacteria. **(B)** OR rats had higher amounts of Clostridia, Bacilli, and Verrucomicrobiae while OP rats had higher Bacteroidia, Actinobacteria, Coriobacteria, and Betaproteobacteria. **(C)** OR rats had higher amounts of orders Clostridiales, Lactobacillales, Verrucomicrobiales, and Turicibacterales while OP had higher amounts of Bacteroidales, Bifidobacteriales, and Burkholderiales. Erysipelotrichi/order Erysipelotrichales was not different between OP and OR rats. **(D)** OR rats had higher amounts of families Lachnospiraceae, Clostridiaceae, Lactobacillaceae, Turicibacteraceae, Peptostreptococcaceae, and Verrucomicrobiaceae while OP had higher Bacteroidaceae, Muribaculaceae (S24-7), Bifidobacteriaceae, Alcaligenaceae, and Ruminococcaceae.

Relative abundances of bacterial taxa after feeding the rats a HFRS were compared based on phenotype (OP vs. OR rats), based on knockdown method (VM, NA, LX, or WA) and also based on the transplant outcome between the two phenotypes compared to the water control transplant (Figure 6). The patterns observed in the alpha and beta diversity analysis were confirmed after taxonomic assignments as more taxonomic groups were observed in OR rats albeit at very low amounts e.g., Class Deferribacteres (Figure 6).

### Firmicutes

Qualitative analysis showed that at phylum level, OR rats had higher relative proportions of Firmicutes; 62.2% in OR rats while OP had 32.6%. Among classes in this phylum OR rats had higher amounts of Clostridia and Bacilli, 40.1 and 10.8%, compared to 17.6 and 3.9% for OP rats, respectively. Proportions of class Erysipelotrichi were similar between OP and OR rats at 13 and 14%, respectively. Breaking down the data at order levels revealed that class Clostridia was composed entirely of order Clostridiales in both OP and OR rats. Class Bacilli was composed of order Lactobacillales and Turicibacterales with OR rats having 9.2 and 1.6%, compared to OP 3.9 and 0.02%, respectively. Families in the order Clostridiales were Clostridiaceae, Lachnospiraceae, Peptococcaceae, and Peptostreptococcaceae. The only family among Firmicutes that was higher in OP rats was Ruminococcaceae at 3.6% in OP compared to 0.7% in OR rats. All other Clostridiales families were present in higher proportions in OR rats. The major difference in proportions was in Clostridiaceae where OR rats had 14.2% compared to OP rats at 0.47%. Lachnospiraceae abundance was higher in OR (26.0%) compared to OP (17.4%). Clostridiaceae was composed mainly of Clostridium cluster I; Clostridium sensu stricto at 8.71% in OR rats while it ranged from 0 to 0.03% in OP rats. Class Bacilli was comprised of families Lactobacillaceae and Streptococcaceae and Enterococcaceae. OR rats had higher proportion of Lactobacillaceae and Streptococcaceae both at 6.94 and 2.2% respectively while OP rats had 3.15 and 0.7% respectively (Figure 6).

### Verrucomicrobia

The phylum Verrucomicrobia contained only a single uncultured *Akkermansia* species (class Verrucomicrobiae, order Verrucomicrobiales family Verrucomicrobiaceae) with 3.55% abundance in OR rats and 0.27% in OP rats. It was notable that OR rats treated with NA antibiotic had lower alpha diversity (Figure 3E) compared to OR rats treated with VM antibiotic, LX and WA. These NA treated rats had no Verrucomicrobia detected but instead had higher amounts of class Coriobacteriia (phylum Actinobacteria) compared to those treated with WA, LX, and VM (Figure 6).

### Bacteroidetes

The phylum Bacteroidetes consisted solely of the class Bacteroidia and order Bacteroidales and was represented by over twice the abundance in OP rats at 47.8% while OR rats had 21.4%. Each of the families under this phylum, i.e., Bacteroidaceae, Muribaculaceae (S24-7), Rikenellaceae and Porphyromonadaceae were present in higher proportions in OP rats compared to OR rats. Specifically, OP rats had 21.9% Muribaculaceae while OR rats had 7.3%. The OP rats had 12.5% Bacteroidaceae while OR had 8.8%. Families Rikenellaceae and Porphyromonadaceae were present in much lower proportion in both OP and OR (Figure 6).

### Actinobacteria

The OP rats had 15.5% of their bacteria in phylum Actinobacteria while OR rats had 6.5%. Classes among this phylum were Actinobacteria (12.1% in OP vs. 4.2% in OR) and Coriobacteriia (3.4% in OP vs. 2.2% in OR). Class Actinobacteria comprised of order Actinomycetales (0.7% in OP vs. 0.2% OR) and Bifidobacteriales (12.1% in OP vs. 3.8% in OR). Class Coriobacteriia was comprised entirely of order Coriobacteriales. Families in this phylum were Bifidobacteriaceae (12% in OP and 3.8% in OR), Coriobacteriaceae (3.2% in OP and 1.2% in OR) and Micrococcaceae at much lower proportions (Figure 6).

### Proteobacteria

Bacteria from phylum Proteobacteria were present at similar proportions in OP and OR rats; 3.5% in OR and 3.8% in OP. However, at class level, Betaproteobacteria was higher in OP at 2.7% compared to 1.3% in OR while class Gammaprotobacteria was lower in OP at 1.7% compared to 2.7% in OR. Betaproteobacteria was comprised entirely of order Burkholderiales with only family Alcaligenaceae detected. Gammaprotobacteria was composed of order Enterobacteriales with only family Enterobacteriaceae detected (Figure 6).

### Linear Discriminant Analysis Effect Size (LEfSe)

**Figure 7:**
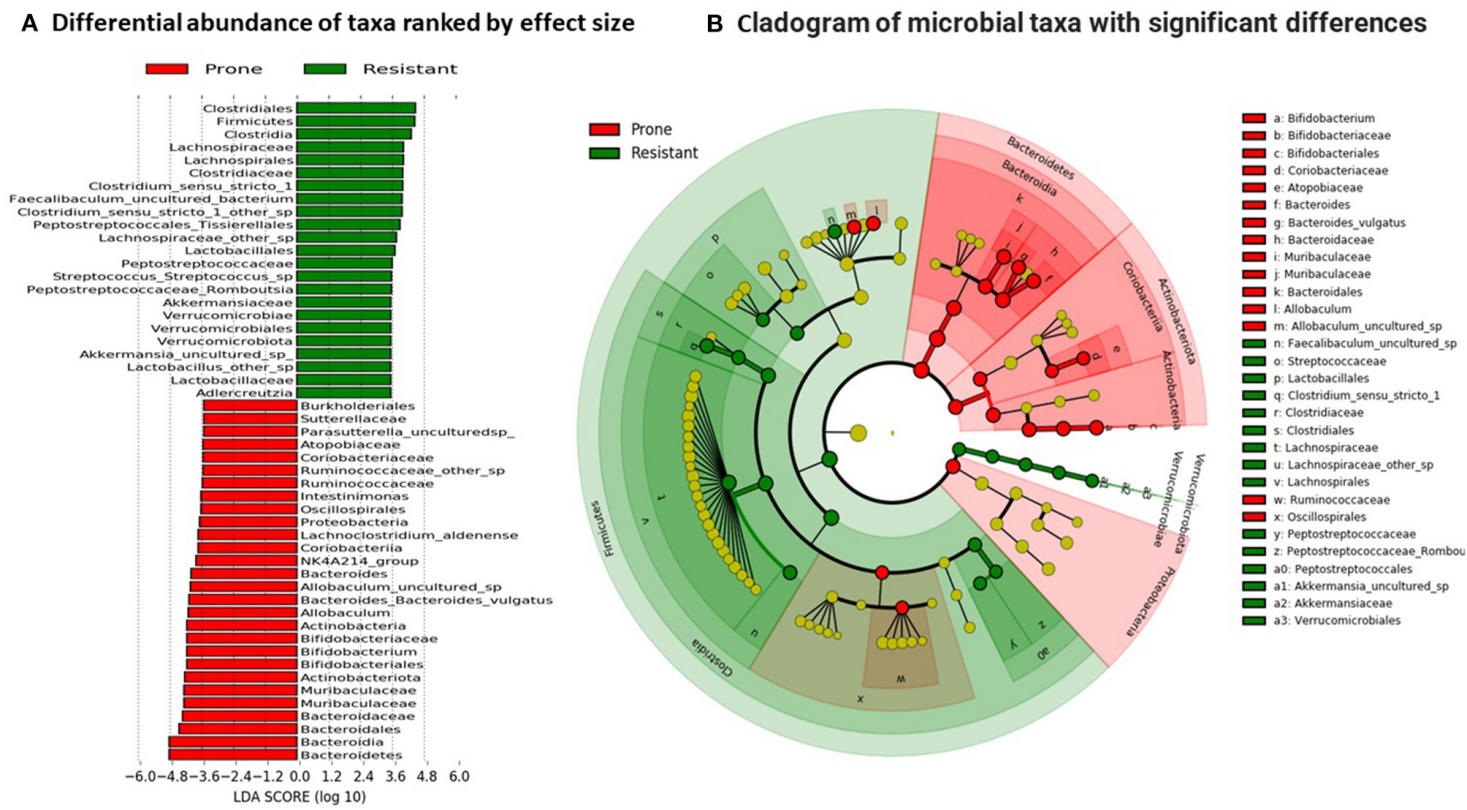
Linear discriminant analysis. **(A)** Differential abundance of taxa was ranked according to their effect size between OP and OR rats. Selection of discriminative taxa between groups were based on an LDA score cutoff of 3.0 and differences in the relative abundances of taxa (converted to log base 10) were statistically determined based on a Kruskal–Wallis and pairwise Wilcoxon tests. A *p*-value of < 0.05 and a score ≥ 3.0 were considered significant in Kruskal–Wallis and pairwise Wilcoxon tests, respectively, at a significance level of 0.05; *n* = 24. The length of the histogram represents the LDA score; i.e., the degree of influence of species with significant difference between different groups. **(B)** The red or green shading depicts bacterial taxa that were significantly more abundant in either the OP or OR groups, respectively. The yellow circles on the cladogram depict bacterial taxa that were not significantly different or changed. Selection of discriminative taxa between groups were based on an LDA score cutoff of 3.0.

The LEfSe algorithm was used to identify the key bacterial taxa that account for the differences between the OP and OR phenotypes. LEfSe estimates the effect size of significantly different abundances of taxa and ranks them represented by the length of each bar (16). At a threshold of 3.0 on the logarithmic LDA score, the abundance of 74 taxa were different between the OP and OR rats and accounted for discriminative features between the rats (Figures 7A,B). A *p* < 0.05 was considered significant in a non-parametric Kruskal-Wallis rank sum test. The LEfSe output was in agreement with abundances shown in the qualitative plots of taxa abundances (Figure 6) showing that order Clostridiales specifically families Clostridiaceae and Lachnospiraceae were most abundant in OR rats while families Bacteroidaceae and Muribaculaceae were most abundant in OP rats.

### Correlation Between ECW, Alpha Diversity, and Abundance in Specific Taxa

The empty cecum weight (ECW) is a marker of fermentation levels in the cecum and was significantly higher in OP rats compared to OR rats after including RS in the diet (*P* < 0.001) (Table 3). We first performed Spearman correlations between ECW and measures of alpha diversity and evenness. ECW negatively correlated with the alpha diversity measures: Shannon index (ρ = −0.61; *P* < 0.001), Number of ASVs (ρ = −0.54; *P* < 0.001), Faith-pd (ρ = −0.51; *P* = 0.001), and Pielou-e index (ρ = −0.56; *P* < 0.002) (Supplementary Figure 2). Higher fermentation was thus associated with lower alpha diversity.

**Figure 8:**
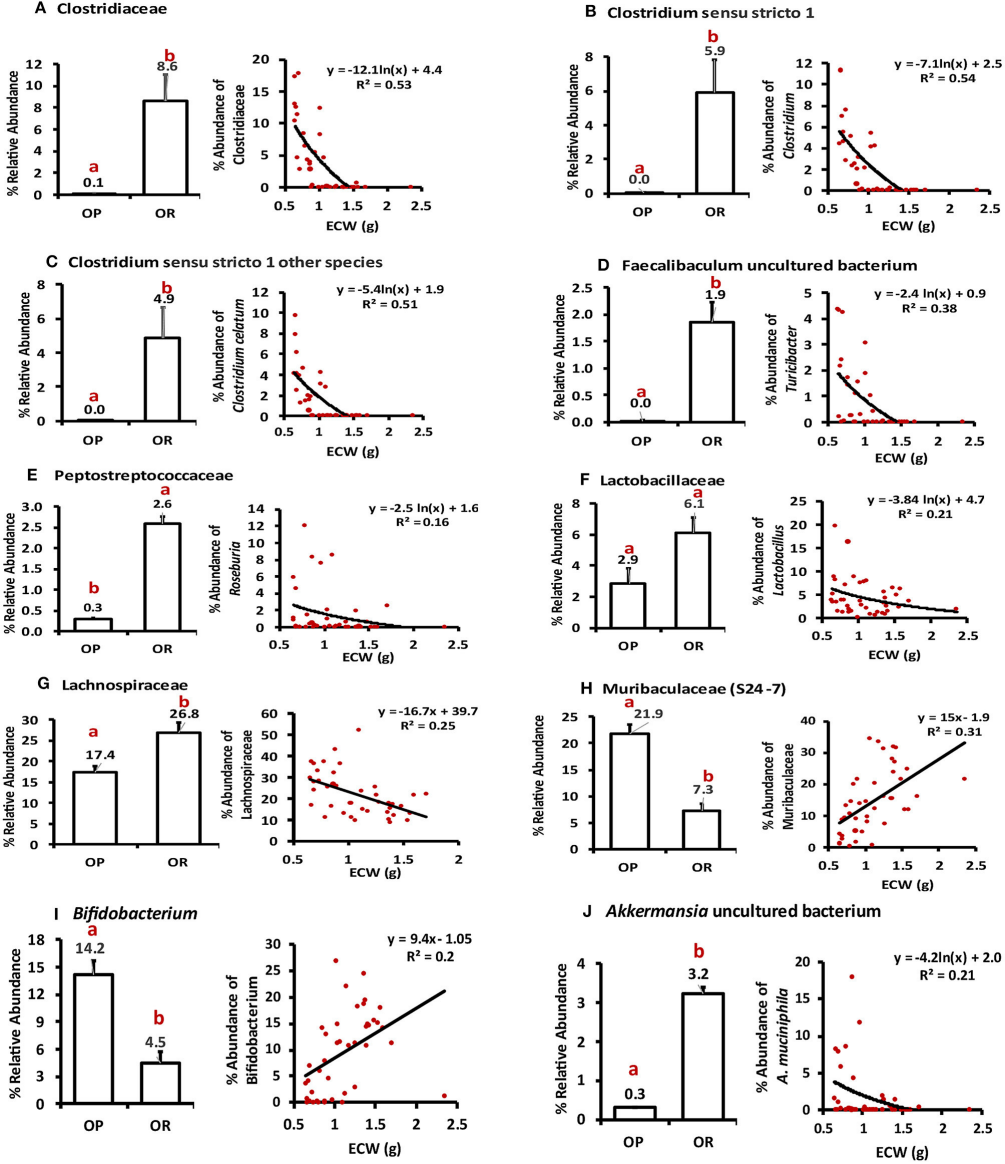
Pearson correlation between ECW and relative abundance of particular taxa. **(A)** ECW negatively correlated with the abundance of family Clostridiaceae (*R*^2^ = −0.55; *P* < 0.001). **(B,C)** Genera under Clostridiaceae *Clostridium sensu stricto 1* and other species in this genus negatively correlated with ECW with *R*^2^ of −0.51 and 0.54; *P* < 0.001). **(D)** Genus *Faecalibaculum* negatively correlated with ECW with an R^2^ of −0.38; *P* < 0.001. **(E–G)** Families Peptostreptococcaceae, Lactobacillaceae, Lachnospiraceae negatively correlated with ECW with *R*^2^ ranging from −0.16 to −0.25; *P* < 0.01. **(H)** Family Muribaculaceae positively correlated with ECW with R^2^ of 0.31; *P* < 0.001. **(I)** The positive correlation with genus *Bifidobacterium* had an R^2^ of 0.20; *P* < 0.001. **(J)** *Akkermansia uncultured sp*. negatively correlated with ECW with *R*^2^ of 0.21; *P* < 0.001. Different letters indicate significant differences after a T-tests.

We next performed correlations between ECW and taxa groups with differential abundance as identified by LefSE. ECW significantly negatively correlated with bacterial taxa that were more abundant in OR rats i.e., the family Clostridiaceae (*R*^2^ = −0.53), genus *Clostridium sensu stricto 1* (*R*^2^ = −0.54), genus *Clostridium sensu stricto* (other species) (*R*^2^ = −0.51), genus *Faecalibaculum* (*R*^2^ = −0.38). A weaker negative correlation was observed between other taxa among Firmicutes including families Peptostreptococcaceae (*R*^2^= −0.16), Lactobacillaceae (*R*^2^ = −0.21) and Lachnospiraceae (*R*^2^ = −0.25). The genus *Akkermansia* also correlated with *R*^2^ = −0.21 (all at *P* < 0.05; Figure 8). On the other hand, ECW correlated positively with the taxa that increased with greater fermentation i.e., family Muribaculaceae (*R*^2^ = 0.31) and genus *Bifidobacterium* (*R*^2^ = 0.2). Only correlations with an *R*^2^ over ± 0.15 are shown in Figure 8. In summary a lean phenotype was associated with abundance of members of class Clostridia and genus *Akkermansia*.

### Predicted Metabolic Functions

**Figure 9:**
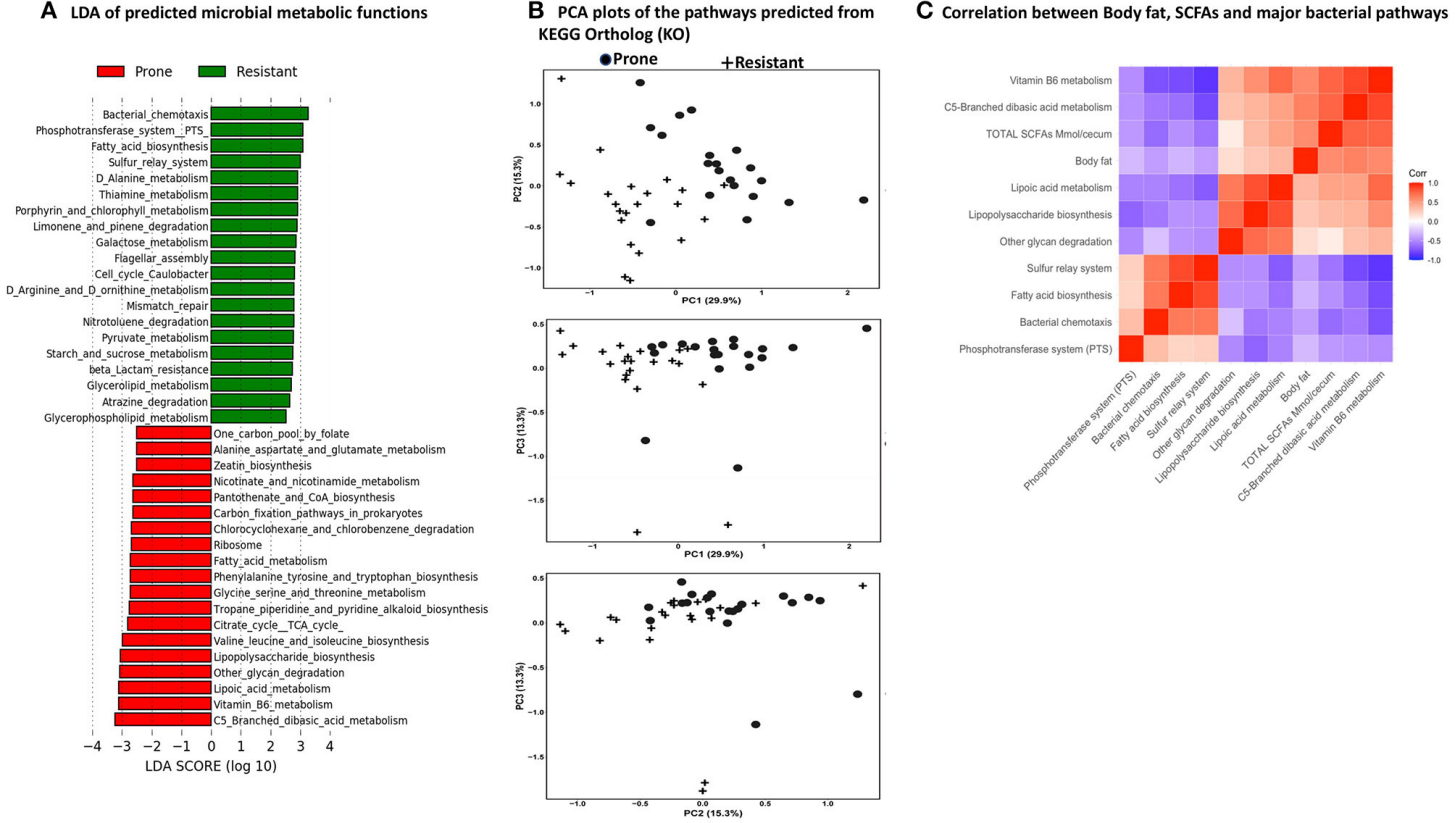
PICRUSt2 Analysis Predicted metabolic pathways by PICRUSt2 using 16s rRNA gene sequences. The pathways were predicted from the Kyoto Encyclopedia of Genes and Genomes (KEGG) Ortholog (KO) database. **(A)** Linear discriminant analysis of predicted microbial metabolic functions between OP and OR rats. Selection of discriminative microbial pathways between groups were based on an LDA score cutoff of 3.0 and differences in the relative abundances of pathway (converted to log base 10) were statistically determined based on a Kruskal-Wallis and pairwise Wilcoxon tests. A *p*-value of < 0.05 and a score ≥ 3.0 were considered significant in Kruskal-Wallis and pairwise Wilcoxon tests, respectively at a significance level of 0.05; *n* = 24. The length of the histogram represents the LDA score; i.e., the degree of influence of species with significant difference between different groups. **(B)** PCA plots to assess beta diversity among the predicted microbial metabolic functions between OP and OR rats. **(C)** Correlation of SCFAs, body fat and major microbial pathways.

PICRUSt2 predicted a total of 152 functional pathways by comparing against KEGG orthologs. Data were transformed to relative abundance and differences between diets are presented in Figure 9. LEfSe analysis at a 3.0 threshold level and at a *p*-value of 0.05 in a non-parametric Kruskal-Wallis rank sum test showed that 38 pathways accounted for discriminative features between the OP and OR rats (Figure 9A). The top 5 pathways significantly higher in the microbiota of OR rats were the bacterial chemotaxis pathway, the phosphotransferase system (PTS), fatty acid biosynthesis, sulfur relay system and D-alanine metabolism. On the other hand the microbiota of OP rats had higher levels of C5-branched dibasic metabolism, vitamin B6 metabolism, lipoic acid metabolism, other glycan metabolism, and lipopolysaccharide (LPS) synthesis. The predicted microbial pathways in OP and OR rats clustered differently after PCA analysis (Figure 9B). Figure 9C shows the visual correlation of amount of SCFAs and body fat with microbial functions.

## Discussion

Because all rats were fed the same diets, this study focused on links between the microbiota rather than diet exposure to the development of obesity. Including both OR and OP rats enabled us to explore a possible link between gut microbiota composition and development of obesity when placed on a high calorie diet and in response to a diet supplemented with resistant starch.

Our results in this study and our previous study (5) demonstrated that when placed on a HF diet OR remain leaner compared to OP rats. We had hypothesized that OR rats would ferment RS to a greater extent than OP rats because our previous research demonstrated that through promotion of fatty acid oxidation rodents fed RS accumulated less abdominal fat compared to those fed the control diet (18, 19). While the results of the current study appear to indicate that greater fermentation is associated with greater obesity, our previous study in which we compared different diets within the two phenotypes, demonstrated that fermentation did not increase abdominal fat compared to a group fed the same diet without RS (with fully digestible starch) (5). Therefore, enhanced fermentation in OP rats does not appear to be a contributing factor to obesity in these rats. We further hypothesized that the difference in susceptibility to accrual of body fat between OP and OR rats may be attributed to the differences in gut microbiota and their predicted metabolic pathways rather than the ability to ferment. Mechanisms attributed to differences in the microbiota would likely have to overcome the effect of increased fat oxidation stimulated by increased fermentation. To test these in the current study, we used a successful knockdown of the bacteria prior to a transplant of the cecal contents between the OP and OR rats. Reduction in bacterial load after antibiotic treatment was confirmed 2 days after the antibiotic regime by qPCR of the 16S gene. The aim was to switch gut microbiota via transplant to increase fermentation in OR rats and decrease it in OP rats to see if the greater obesity would still occur for OP rats. However, there was no change in the difference between the OP and OR rats for fermentation after the transplant. We did not evaluate host genotype or epigenetic differences in this study but it is likely that host genotype dominates diet in shaping the microbiota of these rats. We included MiraLAX in the knockdown treatments to investigate its potential compared to antibiotics which have negative off target effects. However, MiraLAX did not sufficiently knock down the microbiota and effects were not different from that of the water treated control. Analysis of predicted metabolic pathways in gut microbiota revealed that in OP rats the LPS synthesis and glycan degradation pathways were higher than in OR rats and may account for differences in phenotype.

It is possible that the brief exposure of cecal contents to air during collection may have killed strict anaerobic bacterial species and the surviving facultative anaerobic and aero-tolerant bacteria species were not enough to elicit a change in fermentation and lead to a change in accrual of abdominal fat after the transplant. Strict anaerobes play a major role in fermentation and microbiota transplants have been shown to be successful. Satokari et al. (20) used a quick collection of fecal samples to spare strict anaerobes for fecal microbiota transplants (FMT) to treat patients with an overgrowth of pathogenic *C. difficile* bacteria and had a 96% efficacy rate. In another study, Youngster et al. (21) used an aerobic collection procedure and had a 70% efficacy rate. Phenotypic characteristics have been observed to be transferrable in conventionalized rodents after a microbiota transplant under both anaerobic and non-anaerobic conditions (22, 23). The rats in our study were adults at the time of the transplant. In mice it has been shown that the appropriate time-window to perform successful microbiota transfer using non-germ-free animals is shortly after weaning (24). However, a microbiota transplant may be required or found useful at any period of the lifetime to improve microbiota health.

Our conclusion is, the different amounts of bacterial types from one rat phenotype were not as compatible with the genetics and subsequent physiological environment in the GI tract of the different phenotype despite the successful knockdown of bacteria by the two antibiotic cocktails. It is possible that there was resistance to new types of bacteria transplanted from one type of rat phenotype to the other. It appears that once the antibiotic cocktails were completed in the current study, and rats were placed on the diet containing RS, bacterial types were replenished to pre-antibiotic amounts. This is supported by the fact that the difference in fermentation levels between OP and OR were similar to those observed in our previous study (5) that had no knockdown or transplant. In agreement with observations from previous studies (5, 10) robust fermentation of RS was accompanied with increases in Bacteroidetes particularly family Muribaculaceae (S24-7) (5, 10) and Bifidobacterium (23, 25). Muribaculaceae is a member of order Bacteroidales and bacteria in this order are fermentative, but also possess alternative modes of energy production. Carbohydrate-active enzymes constitute about 6% of the Muribaculaceae coding sequences and based on enzyme abundance, the genes encode glycoside hydrolases, largely α-amylases, suggesting starch as a key substrate with the ability to ferment several carbohydrate moieties (24–27). Amounts of Muribaculaculaceae in OP rats were more than double the amounts in OR rats after consumption of RS. This may be an indicator that the GI tract environment of OR rats, both host and microbiota combination, limits the amount of Muribaculaceae (S24-7) and the amount of fermentation.

In agreement with several other studies that show *Bifidobacterium* is enriched during RS interventions (24, 25), OP rats had 14.2% *Bifidobacterium* while OR rats had 4.5% (Figures 6, 8). While *Bifidobacterium longum* was the only species identified in our study, other research works in which *Bifidobacterium* was identified at the species level showed that *Bifidobacterium adolescentis is* the most frequently enriched species with feeding of RS2. It is a primary RS2 degrader, as indicated by its growth on RS2 as a sole carbon source and the ability to adhere directly to α1, 4–linked glucose units within starch granules. These physical interactions likely provide a protective barrier that may explain why RS increases the survival of amylolytic *Bifidobacteria* in the GI tract (24, 25). The differential increase in Muribaculaceae and *Bifidobacterium* lowered alpha diversity in OP rats.

Maintenance of the diversity and collective functional capacity of the bacteria that form the microbiota is vital for optimal metabolic health throughout life. The microbiota of OR rats had overall higher alpha diversity and evenness compared to OP rats (Figures 1, 2) and there was a distinct separation between the microbiota of OR rats and those of OP rats (Beta diversity) (Figures 3–5). Generally, persons and animal models whose microbiota has low gene richness (low bacterial diversity) are more likely to be obese (19, 26) while higher diversity is associated with better health outcomes. We previously observed higher alpha diversity (richness and evenness) in obese ZDF rats fed a control diet with no RS compared to obese ZDF rats fed RS. Lower alpha diversity is a normal and healthy response with the feeding of a relatively high amount of a fermentable fiber. The lower diversity and evenness is due to the enrichment of a subset of bacterial taxa that can efficiently adapt to metabolize the RS polysaccharides and/or degradation products (19, 26). With lower amounts of fermentation, the OR rats had a higher alpha diversity in their gut microbiota. OR rats had a greater abundance of bacteria from phylum Firmicutes specifically Clostridia with family Clostridiaceae and Lachnospiraceae accounting for most differences between OP and OR rats. It was notable that bacteria of the family Clostridiaceae which all belonged to the genus *Clostridium sensu stricto* were not detected in the OP rats but were detected and abundant in OR rats (about 9% of the microbiota) (Figures 6–8). Thus, abundance of Clostridiaceae and Lachnospiraceae were not impacted by dietary RS or its fermentation. The higher representation of Clostridia was accompanied by higher alpha diversity. Members of Clostridia are linked to enhanced immune function. They enhance the development and function of intestinal regulatory T cells (Treg cells) and T follicular helper cells. Treg cells in the intestinal mucosa maintain homeostasis (28–32) and produce large amounts of the anti-inflammatory cytokine IL-10 and protect against obesity, tissue inflammation (32). While previous studies have associated a higher Firmicutes to Bacteroidetes ratio with obesity, our study shows otherwise. The OR rats which had much higher abundance of Firmicutes and therefore a higher Firmicutes to Bacteroidetes ratio, were leaner.

Verrucomicrobia consisting entirely of mucin degrading *Akkermansia sp*. was much more abundant in OR rats; 3.6% in OR rats while OP had 0.3%. The LDA analysis confirmed differentially higher amounts in OR rats and accounts for differences in the two phenotypes. Furthermore, abundance of *Akkermansia sp*. inversely correlated with body weight and an improved metabolic profile. However, it is notable that among OR rats, those treated with NA antibiotic had no Verrucomicrobia detected and had much lower alpha diversity compared to the OR rats treated with VM, LX and WA (Figures 3E, 6). These NA treated rats instead had higher abundance of Coriobacteriia. *Akkermansia sp*. is usually abundant in healthy guts and has been shown to be important in maintaining intestinal integrity and attenuate high-fat diet induced obesity and metabolic syndrome (33–36).

To investigate the relationship between obesity and gut microbiome functions, we predicted the potential metagenomes from the community profiles of 16S rRNA genes using PICRUSt2. The chemotaxis pathway was found to be differentially higher in the microbiota of OR rats. Chemotaxis, a process transduced via protein-protein interactions is a mechanism by which bacteria efficiently and rapidly respond to changes in the chemical composition of their environment toward chemically favorable environments and avoid unfavorable environments. Bacteria continuously monitor a spectrum of sensory inputs. While the OP and OR rats were on the same diets, the sensory inputs may differ in the intestinal environment after fermentation and ultimately impact nutrient uptake and utilization by the bacteria (37). While OR rats had much less SCFAs detected in the intestinal contents, PICRUt2 analysis showed that their fatty acid biosynthesis pathway was significantly higher compared to OP rats. The primary role of bacterial fatty acids is to act as the hydrophobic component of the membrane lipids (phospholipids) and they are also components of storage lipids e.g., polyhydroxyalkanoic acids in several bacterial species (38). While the role of SCFAs in this process is unclear, we found a negative correlation of SCFAs levels with fatty acid biosynthesis pathway (Figures 9A–C). The phosphotransferase system (PTS) a distinct method employed by bacteria for sugar uptake and phosphorylation using energy from phosphoenolpyruvate (PEP) was higher in the microbiota of OR rats. Several monosaccharides, disaccharides, amino sugars, polyols, and other sugar derivatives are consumed by this process. PTS regulation network controls carbohydrate uptake and metabolism and has numerous regulatory functions (39). This may account for why starch and sucrose metabolism pathway was differentially higher in the OR rats (Figure 9A). The polysaccharide (LPS) synthesis, other glycan metabolism and lipoic acid metabolism were significantly higher in the microbiota of OP rats. Evidence has shown that LPS is involved in the development of obesity as a direct targeting molecule for lipid delivery and storage in adipose tissue and is involved in inflammatory mechanisms (40). While the human genome is capable of fully degrading a very small subset of glycans that have only one or two different linkages i.e., starch, lactose and sucrose, bacteria possess the corresponding enzymatic tools for depolymerizing numerous glycans into their component monosaccharides. Gut microorganisms vary widely in the number of different glycans that they can target (41). It is possible that the microbiota of OP rats degrades different types of glycans including larger structures like the plant cell wall or ubiquitously abundant dietary glycans, thus enhancing their capacity to harvest more energy from the host diet and thus promote host obesity. The enhanced lipoic acid metabolism may be as a result of this enhanced glycan metabolism to support energy production and the regulation of carbohydrate and protein metabolism.

The role of host genetics on the obesity phenotype may be greater than the role of the gut microbiota in these rats. Furthermore, host genetics may impact gut microbiota composition and thus explain why the transplant was not successful. Other researchers have previously shown that interactions between gut microbiota, host genetics, and diet modulate the predisposition to obesity and metabolic phenotypes (42–46). Goodrich et al. (42) compared microbiotas across more than 1,000 fecal samples obtained from the TwinsUK population and identified many microbial taxa whose abundances were influenced by host genetics. In a study using eight genetically distinct inbred mouse strains obtained from the same facility and subjected to the same environmental conditions, Krezner et al. (43) showed that the response to a high-fat/high-sucrose diet, was dependent on genetic variance and that host genetics regulated the composition of the microbiota.

In summary, we sought to determine whether a gut microbiota transplant between the OP and OR rats can result in a phenotype change based on acquisition of a changed microbiota. The transplant did not change the microbial composition or phenotype of the OP and OR rats. Based on the current and a previous study with OP and OR rats, other host-microbiota mechanisms besides fermentation may promote their different phenotypes. In addition to microbiota composition, genetic or epigenetic differences between the rats may play a major role in the ultimate phenotype of these rats. The most significant difference in microbiota composition that may promote resistance to obesity in OR rats was the higher representation of the families Clostridiaceae and Lachnospiraceae and genus Akkermansia and the higher alpha diversity. In addition, the different bacterial compositions precipitated in differences in the predicted microbial pathways that may account for the different phenotypes. These predicted pathways include chemotaxis, phosphotransferase system (PTS), and fatty acid biosynthesis in OR rats and glycan metabolism and LPS synthesis in OP rats. Because the microbiota transplant did not result in significant changes in microbiota composition or phenotype, we propose conducting a co-housing study between the two phenotypes as follow-up to monitor microbiota composition and body weight over a specific time frame.

## Data Availability Statement

The datasets presented in this study can be found in online repositories. The names of the repository/repositories and accession number(s) can be found at: https://www.ncbi.nlm.nih.gov/sra/PRJNA746610.

## Ethics Statement

The animal study was reviewed and approved by Louisiana State University Institutional Animal Care and Use Committee (IACUC) as 15-109.

## Author Contributions

DO designed the study, performed animal study, and wrote the manuscript. MK designed the study, performed the animal study, and edited the manuscript. AR performed part of the animal study and tissue analysis. RP performed part of the animal study. CT performed sequencing and bioinformatics. BM advised on and performed statistical data analysis. RS performed part of the animal study. JG performed part of the animal study. DC performed part of the animal study and formulated the diets. CH designed the study, performed bioinformatics, statistical analysis, and edited the manuscript. All authors contributed to the article and approved the submitted version.

## Funding

Research funded by USDA NIFA Hatch Project (Michael Keenan) and Ingredion Incorporated. Starches were gifts from Ingredion Incorporated. Research partially supported by NIH NORC Center Grant #P30DK072476 entitled Nutritional Programming: Environmental and Molecular Interactions. Ingredion Incorporated was not involved in the study design, data collection, analysis, interpretation of data, the writing of this article or the decision to submit it for publication.

## Conflict of Interest

JG was employed by company BIO-CAT. The remaining authors declare that the research was conducted in the absence of any commercial or financial relationships that could be construed as a potential conflict of interest.

## Publisher's Note

All claims expressed in this article are solely those of the authors and do not necessarily represent those of their affiliated organizations, or those of the publisher, the editors and the reviewers. Any product that may be evaluated in this article, or claim that may be made by its manufacturer, is not guaranteed or endorsed by the publisher.

## Abbreviations

AIN-93: American Institute of Nutrition-1993
CD: Cesarean derived
EBW: Emboweled body weight
HF: High Fat
HOMA-IR: Homeostatic model of assessment of insulin resistance
LF: Low Fat
LX: Miralax®
NA: Neomycin, and ampicillin
OP: Obesity Prone
OR: Obesity Resistant
RS: Resistant Starch
VM: Vancomycin and meropenem
WA: Water.
