# Supplementary material for: Gut Microbiota Composition and Predicted Microbial Metabolic Pathways of Obesity Prone and Obesity Resistant Outbred Sprague-Dawley CD Rats May Account for Differences in Their Phenotype
Source: Front Nutr. 2021 Dec 7;8:746515. doi: 10.3389/fnut.2021.746515 (PMC8691123; doi:10.3389/fnut.2021.746515)
Supplement: Supplementary file 1 [file Data_Sheet_1.docx]

| 1. **Rarefaction by number of ASVs (sequence variants per sample in OP Rats)** | 1. **Rarefaction by number of ASVs (sequence variants per sample in OR Rats)** |
| --- | --- |
| 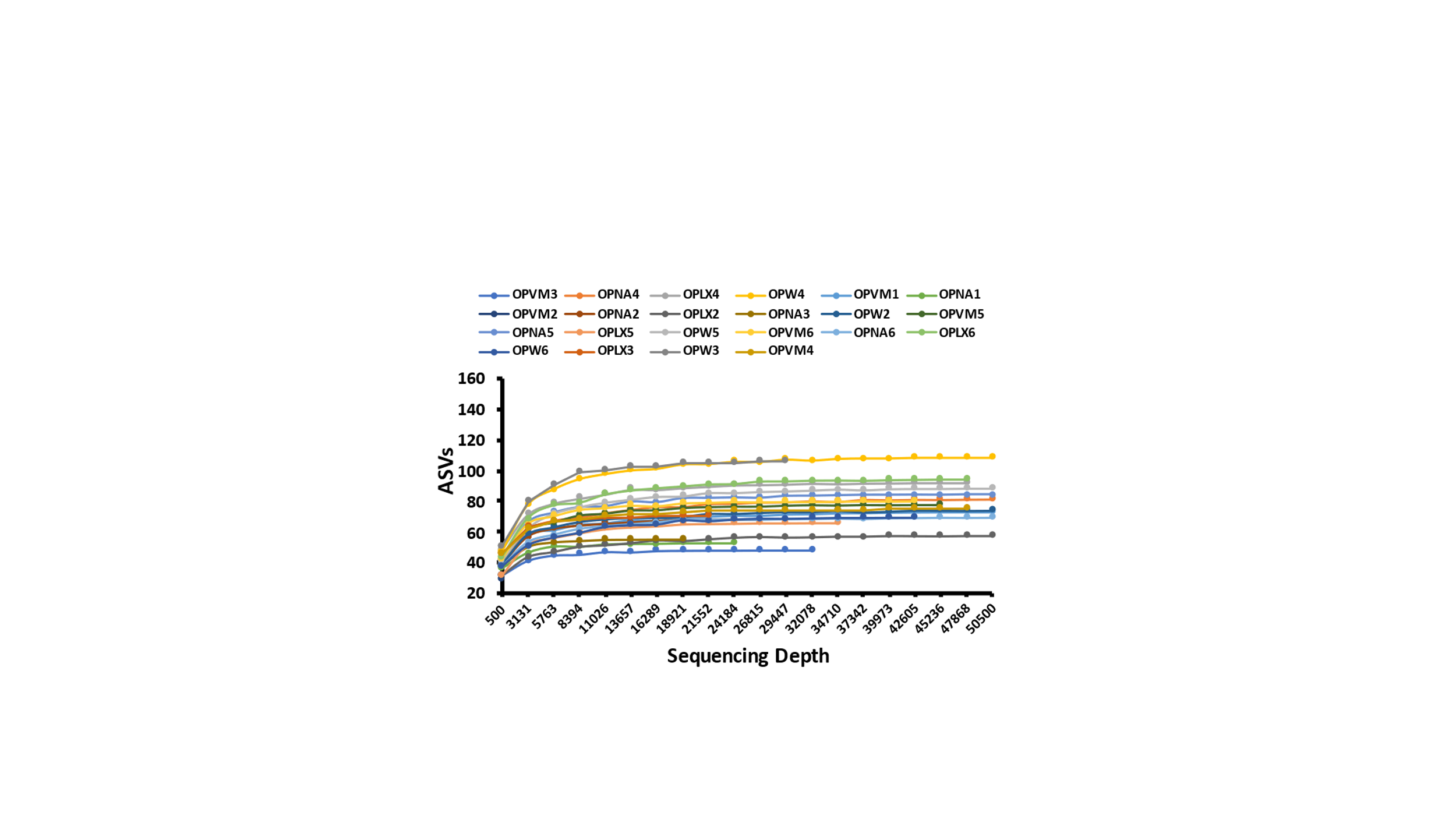 | 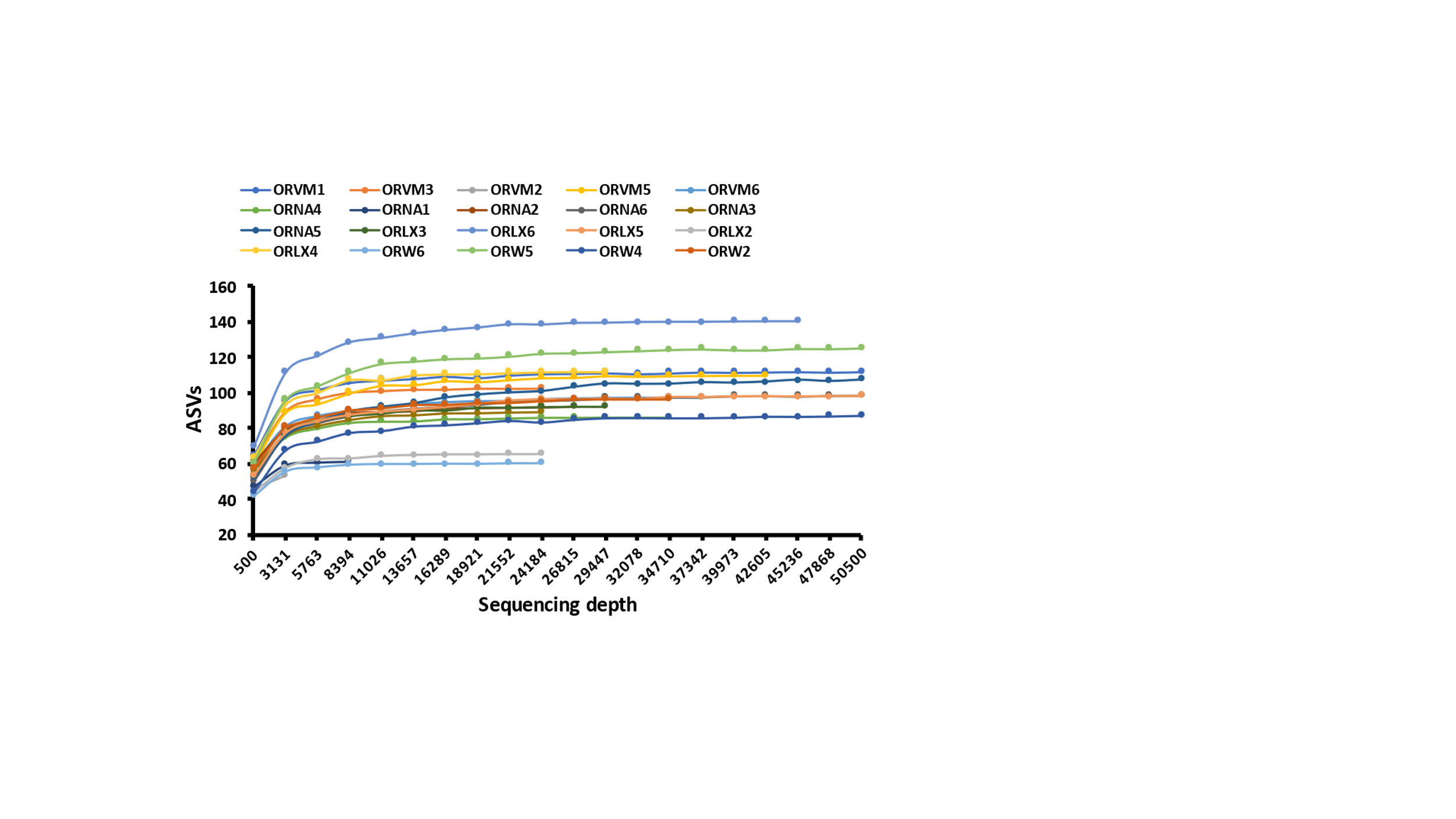 |
|  |  |
| 1. **Rarefaction by Faith-pd in OP rats** | 1. **Rarefaction by Faith-pd in OP rats** |
| 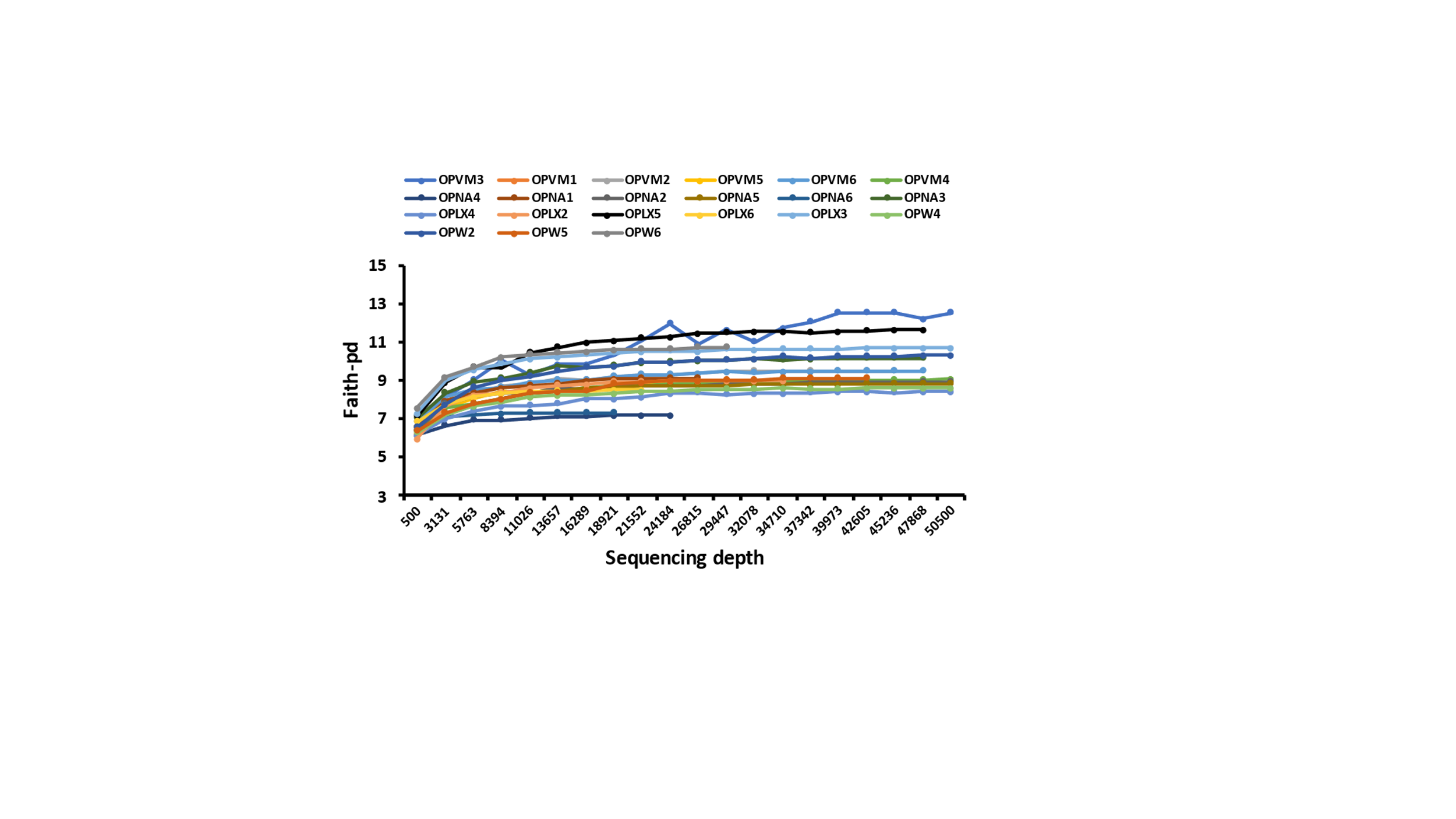 | 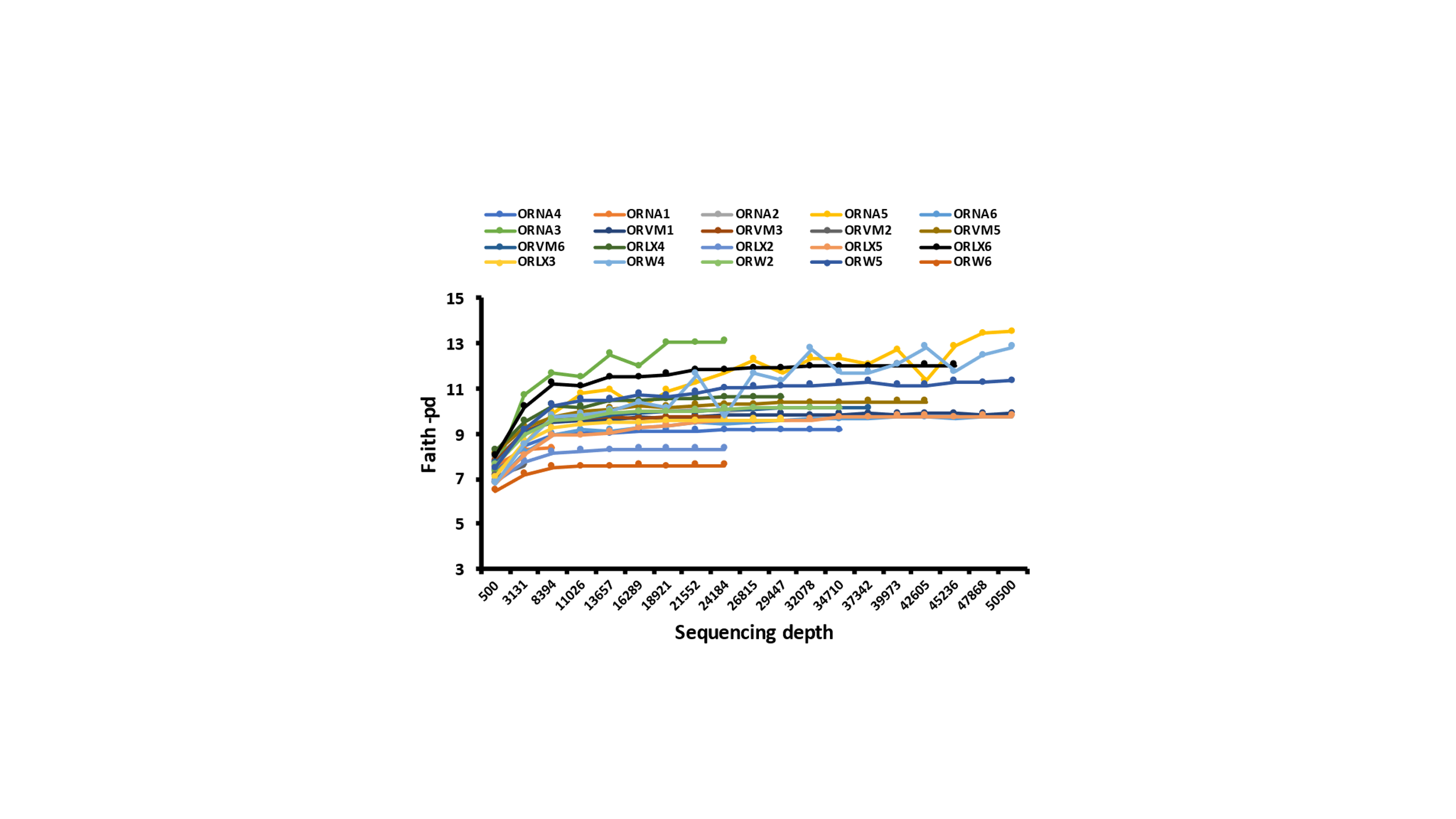 |

**Supplementary Figure 1. Rarefaction curves in each sample**

**(A and B).** Rarefaction based on the number of sequence variants showed richness was higher in OR rats compared to OP rats irrespective of knockdown and transplant. The rarefaction curves for all rats reached a plateau indicating that the sequencing depth was sufficient to detect the majority of ASVs in each sample and capture the microbial diversity.

**(C and D).** Rarefaction based on Faith-Pd showed that alpha diversity taking phylogenetic distance into account was higher in OR rats compared to OP rats irrespective of knockdown and transplant. A moderate sequencing depth (ca. 10,000) was sufficient to capture the majority of the diversity in each group.


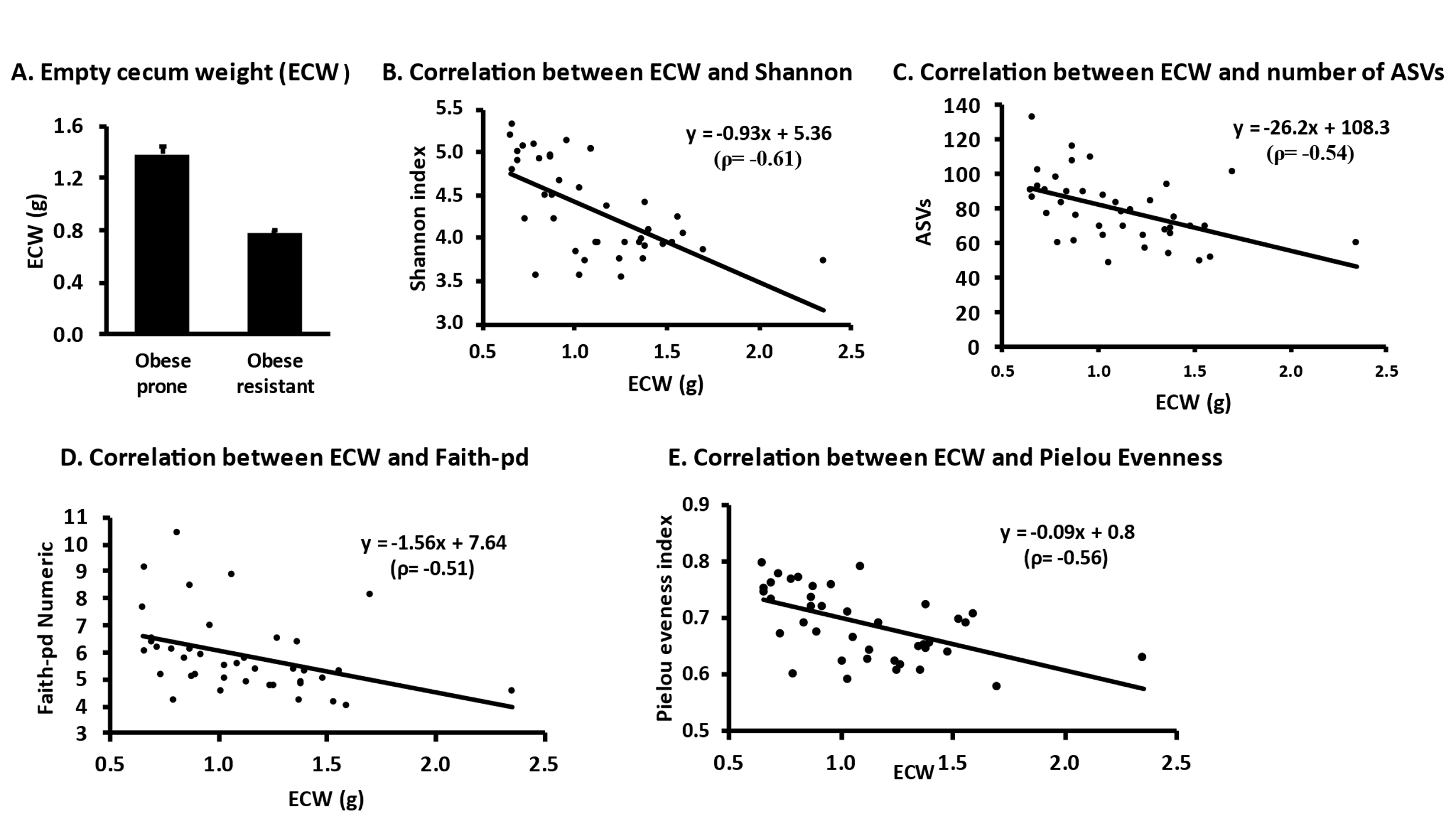


**Supplementary Figure 2. Spearman correletion between ECW and diversity and evenness measures**

**(A).** The weight of the ECW was significantly higher in OP rats compared to OR rats (P<0.001).

**(B)** ECW correlated with the Shannon index (ρ=-0.61; P<0.001).

**(C)** ECW correlated with the numbers of ASVs (ρ=-0.54; P<0.001).

**(D)** ECW correlated with the Faith-pd index (ρ=-0.51; P<0.001).

**(E)** ECW correlated with the Pielou-e index (ρ=-0.56; P<0.001).
